# Oviparous elasmobranch development inside the egg case in 7 key stages

**DOI:** 10.1371/journal.pone.0206984

**Published:** 2018-11-06

**Authors:** Syafiq M. Musa, Molly V. Czachur, Holly A. Shiels

**Affiliations:** 1 Faculty of Biology, Medicine and Health, University of Manchester, Manchester, United Kingdom; 2 School of Environmental and Natural Resource Sciences, Faculty of Science and Technology, Universiti Kebangsaan Malaysia, Bangi, Selangor, Malaysia; Laboratoire de Biologie du Développement de Villefranche-sur-Mer, FRANCE

## Abstract

Embryological stages of oviparous elasmobranch during development can be difficult to identify, requiring magnification and/or fixation of an anaesthetized embryo. These restrictions are poorly suited for monitoring the development of living elasmobranchs inside their egg cases. There are two major aims of this study. The first was to observe elasmobranch embryonic development non-invasively and produce a non-invasive developmental key for identifying the life stages for an elasmobranch inside the egg case. To this end, 7 key developmental stages were identified for the greater spotted catshark, *Scyliorhinus stellaris*, and are provided here with diagrams from multiple perspectives to demonstrate the key features of each stage. The physiological and ecological relevance of each stage are discussed in terms of structure and function for embryonic survival in the harsh intertidal zone. Also discussed is the importance of the egg case membrane and the protective embryonic jelly. The second aim of the study was to understand the applicability of the 7 developmental stages from *S*. *stellaris* to other oviparous elasmobranchs. Thus, changes in embryonic body size and egg yolk volume at each stage were measured and compared with those of the closely related, lesser spotted catshark, *Scyliorhinus canicula*. We find nearly identical growth patterns and yolk consumption patterns in both species across the 7 developmental stages. Thus, although the 7 developmental stages have been constructed in reference to the greater spotted catshark, we suggest that it can be applied to other oviparous elasmobranch species with only minor modification.

## Introduction

Elasmobranchs are a group of cartilaginous fishes from the subclass Elasmobranchii, which consists of sharks, rays, skates, guitarfish and sawfish [1–4]. They can be differentiated from teleost fish (true fish with bony endoskeleton) by several characteristics; their cartilaginous endoskeleton, the possession of five to seven gill openings, rigid dorsal fins or spines, lack of swim bladder, and a body that is covered in denticles or placoid scales [2]. Elasmobranchs are widely distributed globally and exist in high species diversity [1–4]. Elasmobranch species, such as sharks, play an important role in marine ecosystems. They are apex predators and thus are important for ecosystem balance by controlling prey populations [5–7]. A reduction of shark abundance in marine environments suggests that an ecosystem is out of balance [8–10]. Therefore, shark abundance could be used as an indicator of the health of an aquatic ecosystem.

Embryonic development is a vulnerable period during the life cycle of an organism, important for determining survivability and thus influencing population viability [11,12]. Therefore, to fully understand how oviparous elasmobranchs survive their intertidal embryonic life stage, it is necessary to observe and study the development of the embryo inside the egg case. Ballard *et al*. (1993) produced the first complete embryonic developmental table for an elasmobranch [13]. They identified 34 different embryonic stages for the lesser spotted dogfish/catshark, *Scyliorhinus canicula*, under normal developmental conditions (i.e. at 16 °C, with aerated seawater). These stages mark important points in embryological development, but are not easily identifiable with the naked eye or under low magnification, even through cleared egg case membranes. It is not possible to distinguish between the 34 stages without removing the embryo from the egg case. More recently, developmental stages of a batoid oviparous elasmobranch, the clearnose skate, *Raja eglanteria*, have been described and classified based on days of development [14]. Similar to Ballard *et al*. (1993), this study provides excellent embryological detail but the developmental stages are not easily recognizable without specialized knowledge and equipment. In addition, in both studies, the embryo was anaesthetized, euthanized and exposed to fixative for staging [13,14], which is not suitable for studying living, developing embryos.

There is a growing interest in shark conservation leading to aquarists, naturalists and the general public identifying shark egg cases on beaches worldwide. In the UK, organizations such as the Shark Trust (http://www.sharktrust.org/) educate the public in species identification of elasmobranch egg cases, and encourage observations to be recorded in citizen science databases. A user-friendly key that could allow scuba divers, snorkelers, non-embryological researchers and the general public to stage the age of the embryo, may aid elasmobranch monitoring and conservation efforts by providing egg case laying and hatching time to be estimated in the field. Additionally, there is a growing demand for monitoring of elasmobranch development inside the egg cases for use in studies linking development to climate change and metabolism [15–17]. Thus, our study aims to produce a developmental scale which can be used without specialized equipment, and applied non-invasively to living, developing elasmobranch embryos.

We used the oviparous elasmobranch, the greater spotted catshark, *Scyliorhinus stellaris* to construct our developmental stages. About 40% of elasmobranch species are oviparous, predominantly the benthic sharks (family Scyliorhinidae, Hemiscylliidae and Heterodontidae) and all skate species [2,18–20]. *S*. *stellaris* is a benthic catshark from the family Scyliorhinidae [2], is widely distributed and is one of the most abundant elasmobranch species in the British Isles [2,4]. The male *S*. *stellaris* will mature at total body length of ~77 cm, while the female will mature at ~79 cm body length; 162 cm is the longest body length recorded [2,4]. The body has two dorsal fins, where the first dorsal fin is larger than the second, and does not have spines. The whole body is covered with tiny denticles or placoid scales. Body color varies from pale brown to brown and is covered with dark brown to black spots of various sizes on the sides and dorsal surface, whereas the ventral surface is pale yellow to white. They can be found around the continental shelf area of Europe including the Atlantic and Mediterranean Sea at depths ranging from 20–100 m [2,4]. *S*. *stellaris* can be distinguished from closely related species, the small spotted catshark, *Scyliorhinus canicula* by its larger body size and small anterior nasal flaps that do not reach the mouth [2,4]. *S*. *canicula* is one of the most common elasmobranch species in UK waters [2,4] and is the elasmobranch species best studied in terms of embryological development (i.e. used in Ballard’s scale) [13]. Thus, we have also employed *S*. *canicula* in the current study to make reference with earlier embryological works and as a point of comparison with *S*. *stellaris*. Indeed, our aim is to create a parallel scale to the current detailed but invasive embryological scale, that can be used when investigating embryos still remaining in their egg cases.

*Scyliorhinus sps*. lay egg cases (sometimes referred to as mermaid’s purses) in pairs with a single egg case being produced per oviduct [2,4]. *S*. *stellaris* egg cases can be found throughout the year, with the peak breeding season in the spring to summer months [4]. During egg maturation, the gravid females of *S*. *stellaris* swim to the shallow intertidal zone to lay their egg cases in areas with rocky substrate and high vegetation. In this habitat, *S*. *stellaris* egg cases are usually found attached to seaweeds, to substrate with coralline algae [2,21] or associated with other sessile intertidal zone organisms. The intertidal zone is a harsh marine environment with egg cases and their developing embryos encountering fluctuations in temperature, salinity, wave exposure and high predation [2,22–24]. However, question still remain about the developmental adaptations which allow oviparous embryos to flourish in the intertidal zone. Thus, the aim of our study was to produce a simplified developmental scale which could be used without specialized equipment, and applied non-invasively to living embryos. In addition to developing a table of growth in 7 key stages, we have paid particular attention to lesser described features of life inside the elasmobranch egg case including the protective jelly, the opening of seawater slits and other changes in the egg case as they may be key for surviving early in the intertidal zone. Finally, to demonstrate the applicability of our 7 key stages based on *S*. *stellaris* to other oviparous elasmobranchs, and to be able to correlate our non-invasive scale with earlier embryological studies [13], we have examined how each stage identified for *S*. *stellaris* compares with that of *S*. *canicula* in terms of growth and yolk consumption.

## Materials and methods

### Egg cases source and maintenance

All the work contained in this study is on embryos. All protocols including transport, holding conditions and imaging of embryos were approved by the Animal Welfare and Ethics Review Board of the University of Manchester, UK. A total of 46 *S*. *stellaris* egg cases used in this study were supplied by the Native Marine Centre, Isle of Portland, Dorset, UK. All egg cases were laid in captivity from wild caught female adults. The egg cases were usually laid over night, collected in the morning and then shipped in small batches to the University of Manchester, Manchester, UK between November 2015 and August 2016. Additionally, a total of 8 *Scyliorhinus canicula* egg cases were supplied by Ozeaneum, Stralsund, Germany.

Once in the laboratory, the egg cases were transferred into quarantine tanks containing well aerated saltwater (temperature 15 °C, salinity 35 ppt, dissolved oxygen > 95%). Health of the egg cases was checked using ‘candling’ to assess the state of the yolk and in some cases, the embryo. Candling is a method for visualizing the egg yolk and/or the developing embryo as a silhouette by shinning a high intensity light through the back of the egg case.

An egg case was considered healthy if it had an intact, ellipsoid egg yolk located at the centre of the egg case. The jelly inside the egg case, which surrounds the egg yolk and holds it in place, is healthy when it is cleared and transparent. There should be no yolk mass escaping the thin egg yolk membrane, which turns the jelly murky. If an embryo had already developed upon arrival at the University of Manchester, it could be easily identified by the vigorous movement (wiggling) of its tail, the movement of its external gill filaments and the slow and slight movement associated with buccal pumping. A rotten egg case or a dead embryo was easily detected from its bad, rotten fish-like odour and/or lack of embryo movement.

After two days in quarantine, egg cases were transferred to aerated holding tanks (temperature 15 °C, salinity 35 ppt, dissolved oxygen > 95%) incorporating a water filtration system. The temperature, dissolved oxygen, salinity, nitrogen levels and pH of the saltwater were recorded daily. The egg cases were vertically positioned on strings underwater inside the holding tanks such that the egg case opening end pointed downward. Egg cases were tagged with plastic bird ring tags, which have different colors and numbers for individual identification.

### Developmental staging

To observe the developmental stages of *S*. *stellaris*, each egg case was observed three times a week by candling, and morphology and behavior recorded. Most egg cases had a thin, clear egg case membrane (shell/exterior casing) through which the egg yolk and the developing embryo could be easily observed by candling. However, some had a thicker and more strongly pigmented egg case membrane which was gently shaved down using a scalpel blade to create a thinner and more transparent window for observation without cutting deeply or damaging the egg case membrane.

Each embryo was photographed weekly using Canon PowerShot G16. Hand drawings that were digitally colored were made from these photos from multiple perspectives and angles, so that key features of each developmental stage were clearly visible. Each drawing was based on live samples and photos of 5–7 different individuals at the same developmental stage. Original photos for each drawing are supplied in the supporting files.

During development, the seawater slits of the egg case open and the developing embryo is naturally exposed to saltwater. At this stage, 12 embryos were carefully removed from their egg cases and transferred into clear, permeable containers (artificial egg cases) where the oxygenated saltwater could freely move in and out of the container. This allowed morphological details of the later stages of embryonic development to be documented in detail to facilitate comparison between the features of our 7 key stages and those identified in embryological studies (i.e. Ballard *et al*. 1993). For earlier developmental stages, where the seawater slits were not fully opened, a total of 5 embryos were euthanized by using lethal dosage 0.5 g L^-1^ Tricaine methanesulfonate (MS-222), buffered with 0.5 g L^-1^ sodium bicarbonate, removed from the egg cases and photographed. By doing this, each developmental stage could be drawn in detail from different perspectives, showing all the important key features. The higher level of detail was required to make a side-by-side comparison between our non-invasive scale and Ballard’s embryological scale. However, we must emphasize that removal of the embryo from the egg case is not required for our 7 key developmental stages. The 7 key stages were specifically chosen as they are recognizable by the naked eyes via candling, and because they are ecologically and physiologically important to metabolism, growth and thus survivability.

### Growth pattern and average daily body length gain (ADL)

A total of 8 *S*. *stellaris* egg cases were used to examine the growth pattern of the species across the 7 developmental stages. Each embryo was photographed weekly using Canon PowerShot G16, and total body length (cm) was measured weekly using ImageJ software (http://imagej.nih.gov/ij). The mean ± standard deviation (SD) of total body length (n = 8) were calculated to study the growth pattern of *S*. *stellaris*.

Eight *S*. *canicula* egg cases were also used to study the average daily body length gain (ADL) at each of the 7 developmental stages to test the applicability of our staging scale in two different species. ADL (cm day^-1^) was calculated as, ADL = (final TL at given stage—initial TL at the same stage)/duration (in days) of same developmental stage; where TL is total embryo body length (measured from tip of snout to tip of the tail), and stage is a given stage between stage 1 and stage 7. ADL measurements (in cm day^-1^) were also converted to percentages to aid comparison of ADL patterns between *S*. *stellaris* and *S*. *canicula* at each developmental stage.

### Yolk consumption rate

Using the same photographs as above, length, width and depth (in cm) of the external yolk sac were measured weekly using ImageJ software (http://imagej.nih.gov/ij) to calculate the volume (V) of the external yolk sac as, V = (4/3)πLWD; where L, W and D are (1/2)length, (1/2)width and (1/2)depth of egg yolk (in cm) respectively. From this, yolk consumption (YC) rate (cm^3^ day^-1^) across different developmental stages was calculated for *S*. *stellaris* and *S*. *canicula* (n = 8 for each species) as, YC = (initial V at given stage—final V at same stage)/duration (in days) of same developmental stage. Yolk consumption rate measurements (in cm^3^ day^-1^) were also converted to percentages to compare the yolk consumption rates between *S*. *stellaris* and *S*. *canicula* at each developmental stage.

### Development time (embryo age and the duration of each developmental stage)

Age of *S*. *stellaris* and *S*. *canicula* embryos (n = 8 for each species) at different developmental stages was calculated by summing development time (in weeks) starting from lay (week 0) until any given stage, or hatch. Development time was also converted to percentages to compare the age of embryos from each species at each developmental stage and at hatch. Duration of development time for each stage was calculated by summing the total time (in weeks), where the embryo was still in a given developmental stage. This was also converted to percentages to compare the development time in each stage between *S*. *stellaris* and *S*. *canicula*.

### Hatching process

Once the embryos reached their final developmental stage, behavior was closely monitored to study the hatching process. Videos of hatching behavior were recorded using Canon PowerShot G16 and GoPro HERO4 Session. Details of the hatching process were illustrated step-by-step. Once hatched, whole animal wet mass (± 0.01 g) was recorded.

### Effect of air exposure on egg cases

Because elasmobranch egg cases are often washed up onto beaches where they are exposed to air and high temperatures, we additionally wanted to understand the function of the egg case jelly and the egg case membrane in embryo survival. A total of 6 egg cases were identified as unfertilized eggs (egg cases contained unfertilized egg yolks and filled with jelly). These egg cases were removed from water and aerially exposed (at room temperature). Photographs were taken every hour for 50 hours. These photographs were used to measure egg cases size (in cm^2^) using ImageJ software (http://imagej.nih.gov/ij) to estimate the rate of shrinking, and therefore dehydration, over time. As the sharks hatched, 7 empty egg cases were used to measure the desiccation rate of the egg cases without the jelly by using the same method as described earlier. The tendrils of the egg cases were stretched out to their maximum length before they were measured using a measuring tape (± 0.01 cm) before the egg cases were dried.

### Statistical analysis

Linear regressions, correlations and R^2^ coefficient for embryo growth and yolk consumption were calculated. Statistical significance was assessed using one-way repeated measures analysis of variance (ANOVA), followed by Tukey’s multiple comparisons post-hoc test (P < 0.05). Percentage data between species was compared using Mann-Whitney U test (P < 0.05). Details are provided in each figure legend. All statistical analysis was calculated and graphed using GraphPad Prism 7.

## Results

### The egg case

An elasmobranch egg case consists of the egg case membrane (shell/casing), jelly, an egg yolk and the developing embryo. The *S*. *stellaris* egg cases were oval to rectangular in shape with colors ranging from yellowish green to brownish green when wet, to darker shades of the same colors when dry. The egg case mean length was 11.58 ± 0.33 cm, mean width 4.32 ± 0.17 cm and mean height 2.88 ± 0.22 cm (n = 12) when wet. The incubation period of the fertilized egg cases varied depends on water temperature with an average incubation period at 15 °C of 27.25 ± 0.89 weeks (n = 8), and a range of 26 to 29 weeks.

**Fig 1:**
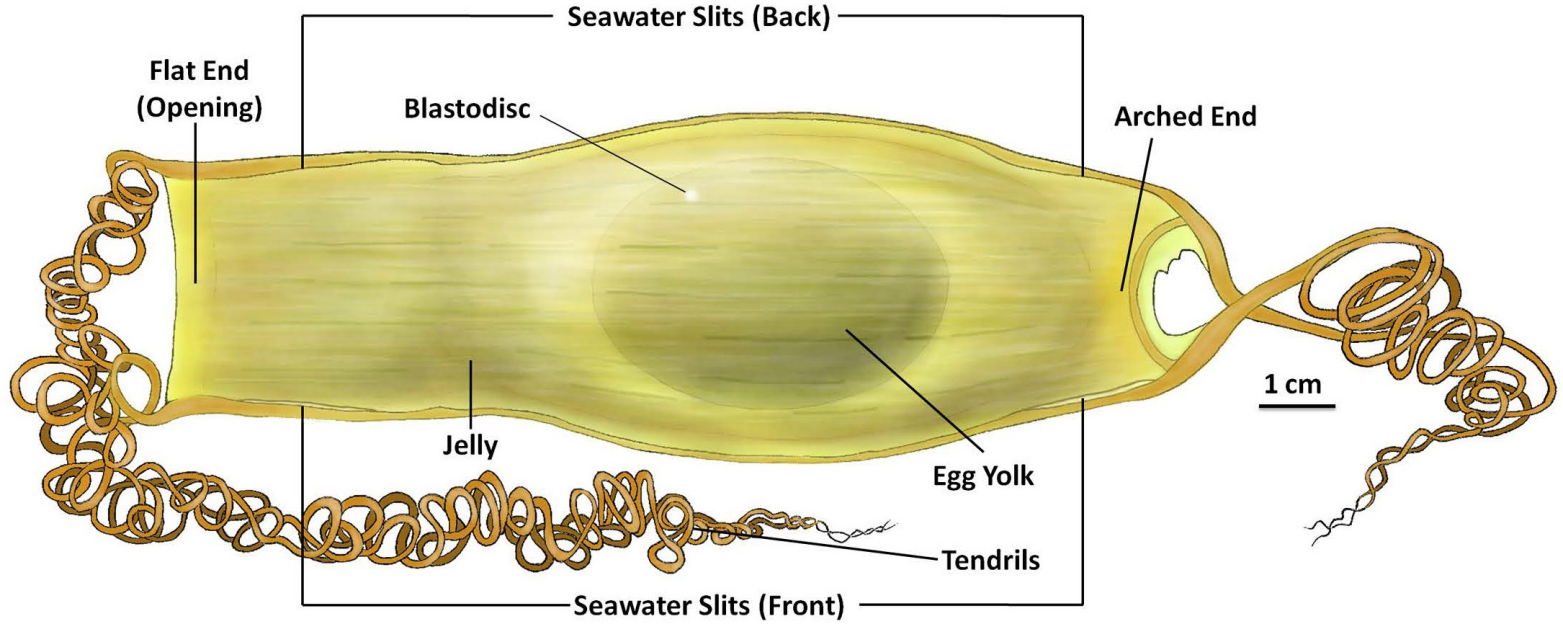
External features of the *S*. *stellaris* egg case at stage 1. The internal ellipsoid egg yolk and blastodisc visible at stage 1 of development. The egg case membrane was filled with jelly surrounding the yolk. There were four seawater slits located at each corner of the egg case, two can be seen from the front (as shown in Fig 1), and another two at the back of the egg case (not shown). The flat end of the egg case will be the site of opening during hatching, whereas the arched end remains firm and closed during hatching. There are four tendrils attached at each corner of the egg case. The key feature for stage 1 was no visible embryo on top of the egg yolk. See S1 File for original photographs of Fig 1 illustration.

The *S*. *stellaris* egg case ends were distinguishable due to one having a flatter edge and the other a more rounded and arched end (Fig 1). The egg cases consisted of four long, spring-like tendrils, located on each corner of the egg case. Tendril mean length was 153 ± 3.98 cm (n = 10) when wet. The tendrils were thicker where they joined the egg case and gradually became thinner towards the ends. The collagenous egg case membrane was flexible but not elastic, so it could easily bend but not stretch. The egg cases had a smooth surface with a leathery to rubbery-like texture. At all edges of the egg case membrane, except the flat egg case opening, there was a much tougher structure present that allowed the egg case to maintain its shape and rigidness.

During the early developmental stage of *S*. *stellaris*, there was an ellipsoid, yellowish egg yolk which was surrounded by a jelly-like substance. This jelly was softer closest to the egg yolk and the developing embryo, and had a tougher consistency as it approached the edges of the egg case. Each *S*. *stellaris* egg case had a total of four seawater slits, which were located towards all four corners of the egg case membrane. These seawater slits could only be found on one side of the egg case, when the egg case was positioned as in Fig 1. These seawater slits were open but blocked by the mucous plugs and the tough jelly, which tightly held the egg case closed from the inside. Due to this, seawater was unable to freely enter into the egg case during the early developmental stages.

### Stage 1

All *S*. *stellaris* egg cases began in stage 1. At stage 1, there was an ellipsoid egg yolk and no visible embryo on top of the egg yolk membrane; this can be observed through the egg case with candling (Fig 1). If there was still no visible embryo on the egg yolk after four weeks, the egg case was usually unfertilized and therefore did not develop further. Also possible at this stage are freshly laid, fully sealed egg cases with no egg yolk visible inside, known as ‘wind eggs’. We observed 3 wind eggs out of 88 *S*. *stellaris* egg cases in this study.

During stage 1, it was necessary to monitor the egg cases closely and more frequently as the embryo developed rapidly. During this stage, the blastodisc can be seen as a small spot, white to light yellow in color, which can be seen on the top of the egg yolk membrane (Fig 1). Many important, but not visible, developmental processes are occurring during this stage, which outlined in the discussion.

Some egg cases had thin and translucent outer egg case membranes, where the blastodisc was clearly visible, whereas some individuals had thicker or more pigmented outer egg case membranes, and the blastodisc was more difficult to locate. This pigmented outer layer could be carefully shaved to create a translucent inner layer window, so that the blastodisc and embryo development could be more readily observed. The position of the blastodisc was influenced by the egg case positioning as it rotated and changed against the gravitational force. This has been described previously for *Scyliorhinus canicula* egg cases [13,25].

### Stage 2

**Fig 2:**
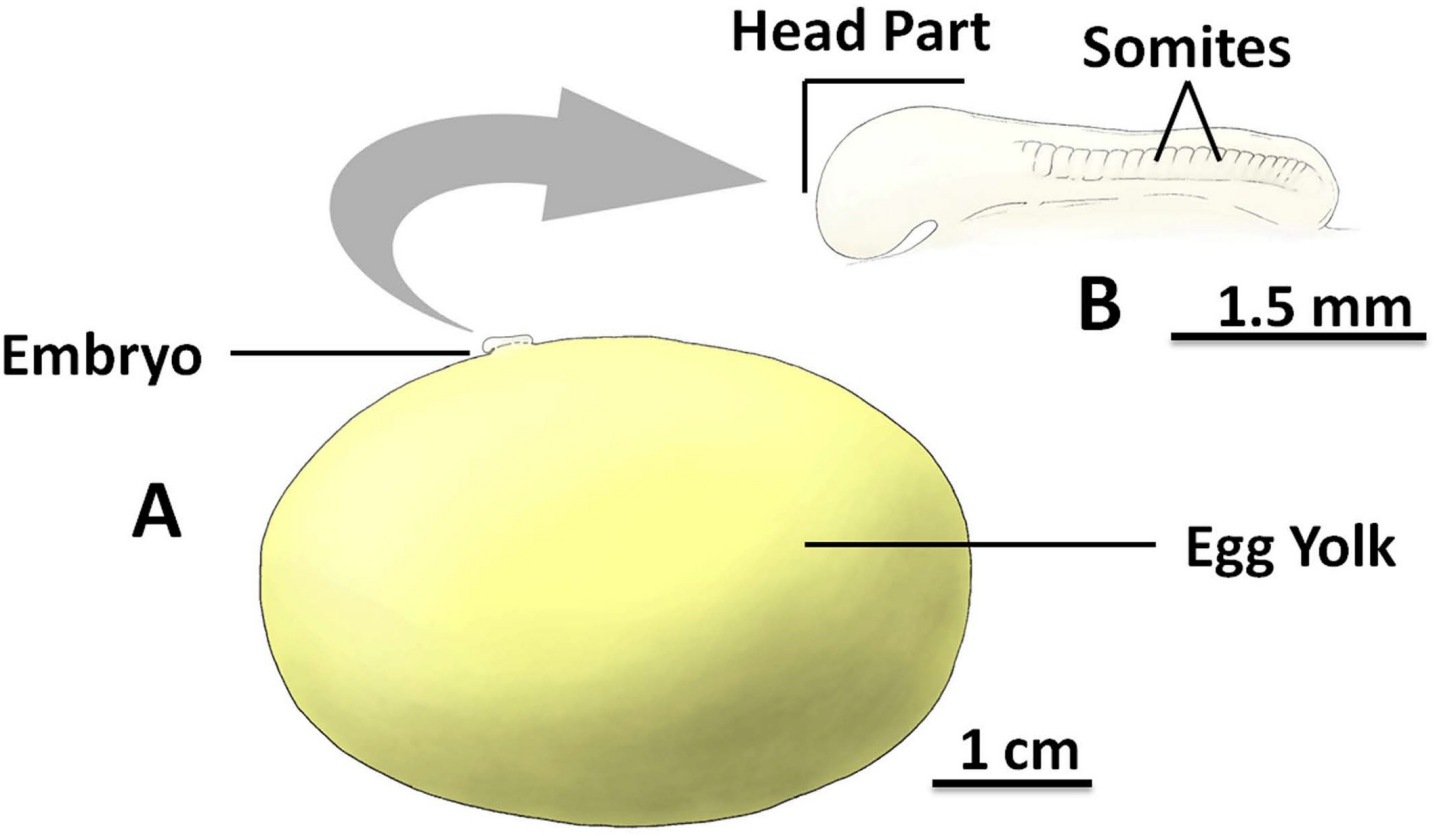
The inside of the *S*. *stellaris* egg case at stage 2. A: The egg yolk mass and the associated embryo to scale. B: The magnified embryo with indication of key morphological features (lateral view). The embryo consists an anterior (head and trunk primordia) and posterior (tail bud), where the somites (segmented mesoderm) can be found. The key feature for stage 2 was the visible embryo developed on top of the egg yolk membrane without a long tail. See S2 File for original photographs of Fig 2 illustrations.

The key feature of stage 2 that can be observed non-invasively through the egg case was the small embryo which was completely connected to the membrane of the ellipsoid-shaped egg yolk (Fig 2). At ~3 weeks old (at 15 °C), embryos reached stage 2 and the total body length ranged from 0.31 cm to 0.98 cm, with mean length of 0.62 ± 0.2 cm (n = 8). As gastrulation continues (see discussion under early embryogenesis for details), the stage 2 embryo looked like an embedded membrane located on the surface of the egg yolk membrane. At this stage, the embryo had two major distinguishable prenatal body regions, the larger sized anterior region, which later develops into the head part and trunk primordia, and the smaller sized posterior region (tail bud), which later develops into the tail of the embryo (Fig 2B). Movement of the embryo could be observed, including regular movements in the head-end of the body from right to left.

There were many somites (segmented mesoderm) on the body towards the posterior of the embryo (Fig 2B), which were difficult to identify or count through the egg case due to the small size and the movement of the embryo. The embryo could also be difficult to locate in the earlier phases of stage 2, as it could be completely flat against the surface of the egg yolk. Similarly to the blastodisc in stage 1, the embryo was most commonly found on top of the egg yolk surface against the gravitational pull. If not in this position, slowly rotating the egg case while searching for the embryo improved the chance of identification.

### Stage 3

**Fig 3:**
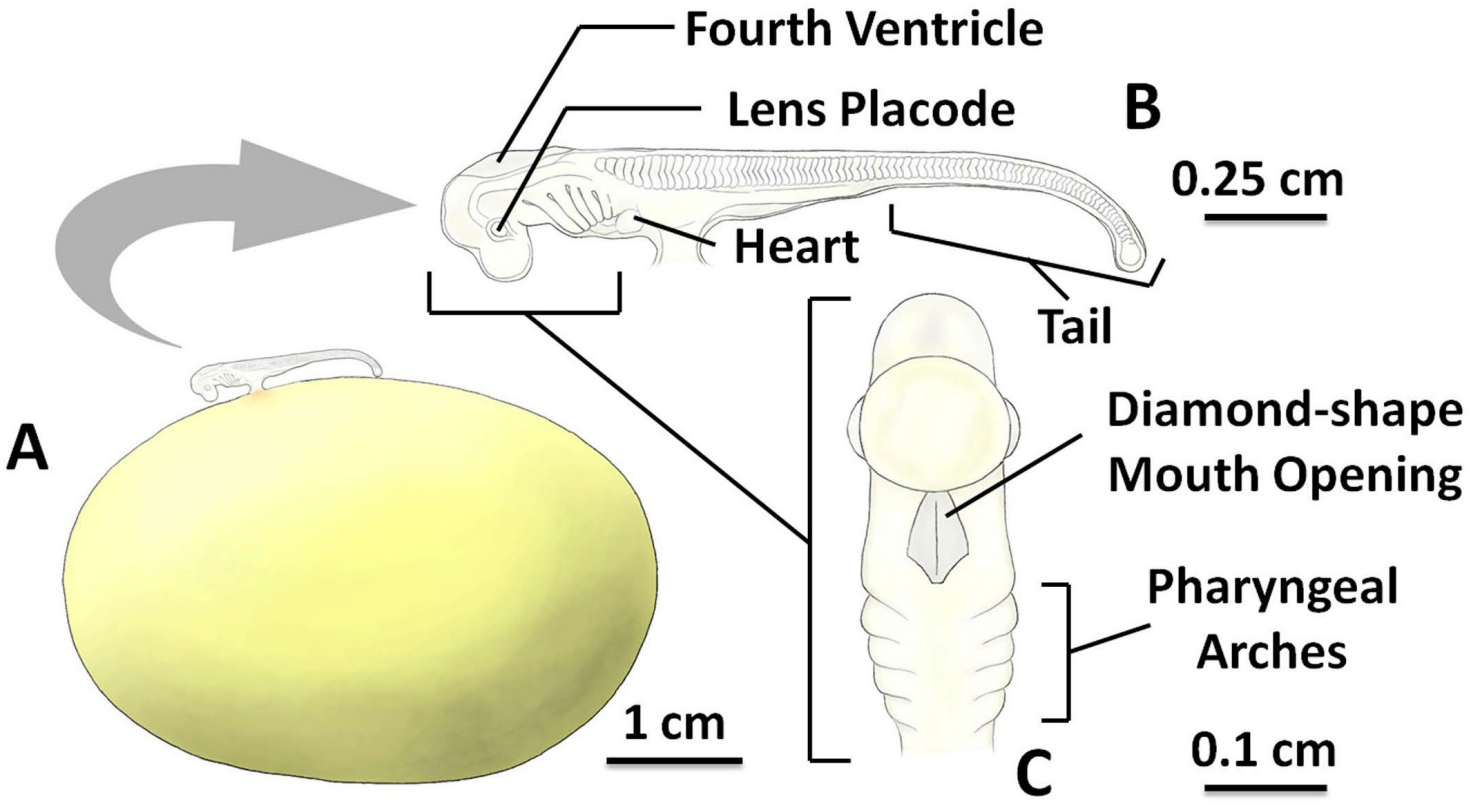
The inside of the *S*. *stellaris* egg case at stage 3. A: The ellipsoid egg yolk mass and the associated embryo to scale. B: The magnified embryo with indication of key morphological features (lateral view). C: The diamond-shape mouth opening (ventral view). The key feature for stage 3 was the growth of the long tail. See S3 File for original photographs of Fig 3 illustrations.

Stage 3 of embryonic development occurred ~4 weeks after laying at 15 °C, and was characterized by the development of long tail (can be observed non-invasively through the egg case) and more than 60% of the embryo body is not connected to the surface of the egg yolk membrane (Fig 3). The embryo was connected to the external yolk sac by the short, wide developing yolk stalk (Fig 3A). The total body length of stage 3 embryos ranged from 0.97 cm to 3.39 cm, with mean length of 1.17 ± 0.16 cm (n = 8). At this stage, most of the organs and body parts began to develop and more somites were visible. The heart is known to have formed [13], but it was still difficult to directly observe through the egg case membrane at this stage as it is small and colorless.

Early stage 3 embryos exhibited a blunt, bent tail tip which pointed downward. Throughout stage 3, the tail became sharper, more slender and straighter as it grew longer. In addition, as the tail grew longer, the tail movement inside the egg case increased. A pair of lens placodes (located in the eye cup), were visible in stage 3 (Fig 3B). The fourth ventricle (located in the brain) was also visible, resembling an empty space on the top of the head. 3–6 pairs of pharyngeal arches (later to support gills) appeared slowly and gradually throughout stage 3. When embryos were viewed ventrally (Fig 3C), it was possible to identify a diamond-shaped opening (later to be the mouth).

At the end of stage 3, small buds of gill filaments begin to develop and located between the pharyngeal arches. The top of the external yolk sac, close to the yolk stalk, changed color from orange to red as blood vessels developed. These blood vessels grew larger and longer throughout stage 3.

### Stage 4

**Fig 4:**
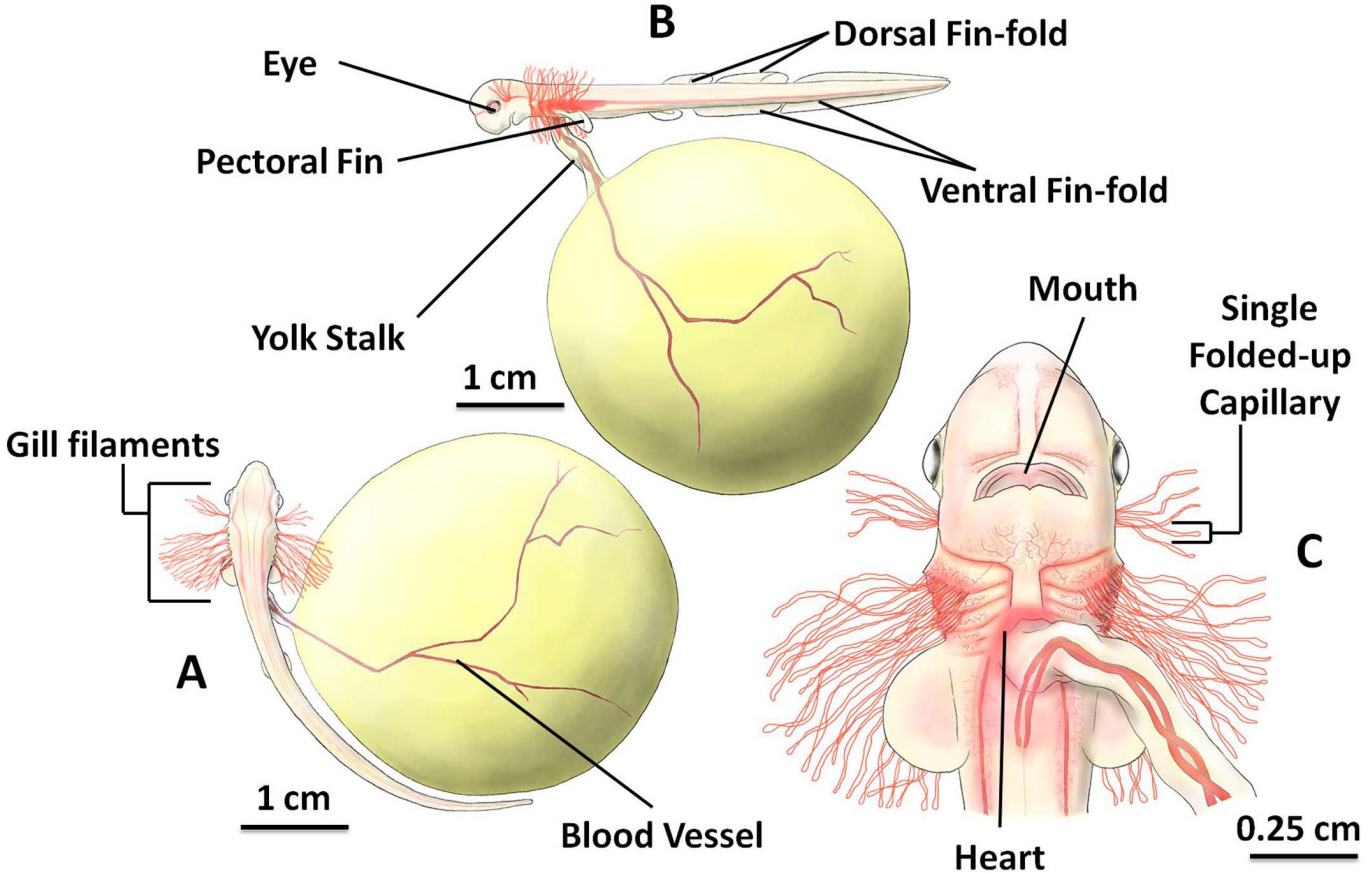
The inside of the *S*. *stellaris* egg case at stage 4. A: Six pairs of filament groups, and more apparent blood vessels on surface of spheroid external yolk sac (dorsal view). B: Developing yolk stalk, eye, and fins (lateral view). C: The magnified head and gill filaments (ventral view). The key feature for stage 4 was gill filament development. See S4 File for original photographs of Fig 4 illustrations.

Between 7 and 10 weeks of age (mean 8.13 ± 0.99 weeks, n = 8) at 15 °C, the embryo reached stage 4 characterized by clearly visible long, red external gill filaments, which can be observed non-invasively through the egg case (Fig 4). Gill filaments length was dynamic; they grew in the early phase of stage 4 and began to shrink near the end of this stage. Total body length ranged from 3.24 cm to 8.32 cm, with mean length of 3.64 ± 0.28 cm (n = 8). Embryo length often exceeded the length of the external yolk sac (Fig 4B), and long gill filaments were arranged into six pairs of filament groups. One pair of the shorter gill filaments were located in the developing spiracles, while the other 5 pairs of longer gill filaments (later to be gill arches) were horizontally positioned, located between the pharyngeal arches (Fig 4A). When closely observed, at this stage each gill filament consisted of a single blood capillary that had been folded up (Fig 4C).

The embryo developed a pair of pectoral fins, a pair of small pelvic fins and numerous large and long fin-folds during stage 4 (3 dorsal fin-folds, later develop into two dorsal fins and the dorsal caudal fin; and 2 ventral fin-folds, later develop into the anal fin and the ventral caudal fin) (Fig 4B). The mouth became more rounded in shape (Fig 4C) and dark pigment was observed in the eye. As more blood vessels developed, the embryo body surface color changed from translucent to pinkish and the skin was thin and non-pigmented. The heart could clearly be seen beating through the body wall from ventral view (Fig 4C) and variable color intensity occurred (flashing of red) as the heart beat propelled blood. It was possible to measure heart rate at this stage from counting changes in color intensity and we calculated resting heart rate at 15 °C to be ~20 bpm.

The shape of the external yolk sac changed from ellipsoid to spheroid during stage 4. The yolk became surrounded by many long blood vessels and the yolk stalk grew longer and narrower as the embryo grew larger in size. By the end of stage 4, the amount of yolk mass that had been transferred into the embryo was less than 28.05 ± 9.03% (n = 8) of the total egg yolk volume at stage 1. Although, the embryo had started to consume the egg yolk, the shrinking of the external egg yolk was not easily appreciable during this stage. At the end of stage 4, more jelly was degraded from the inside of the egg case by the embryo activity, and all four seawater slits were fully opened, exposing the embryo to the surrounding seawater, which freely circulated into and out of the egg case.

### Stage 5

**Fig 5:**
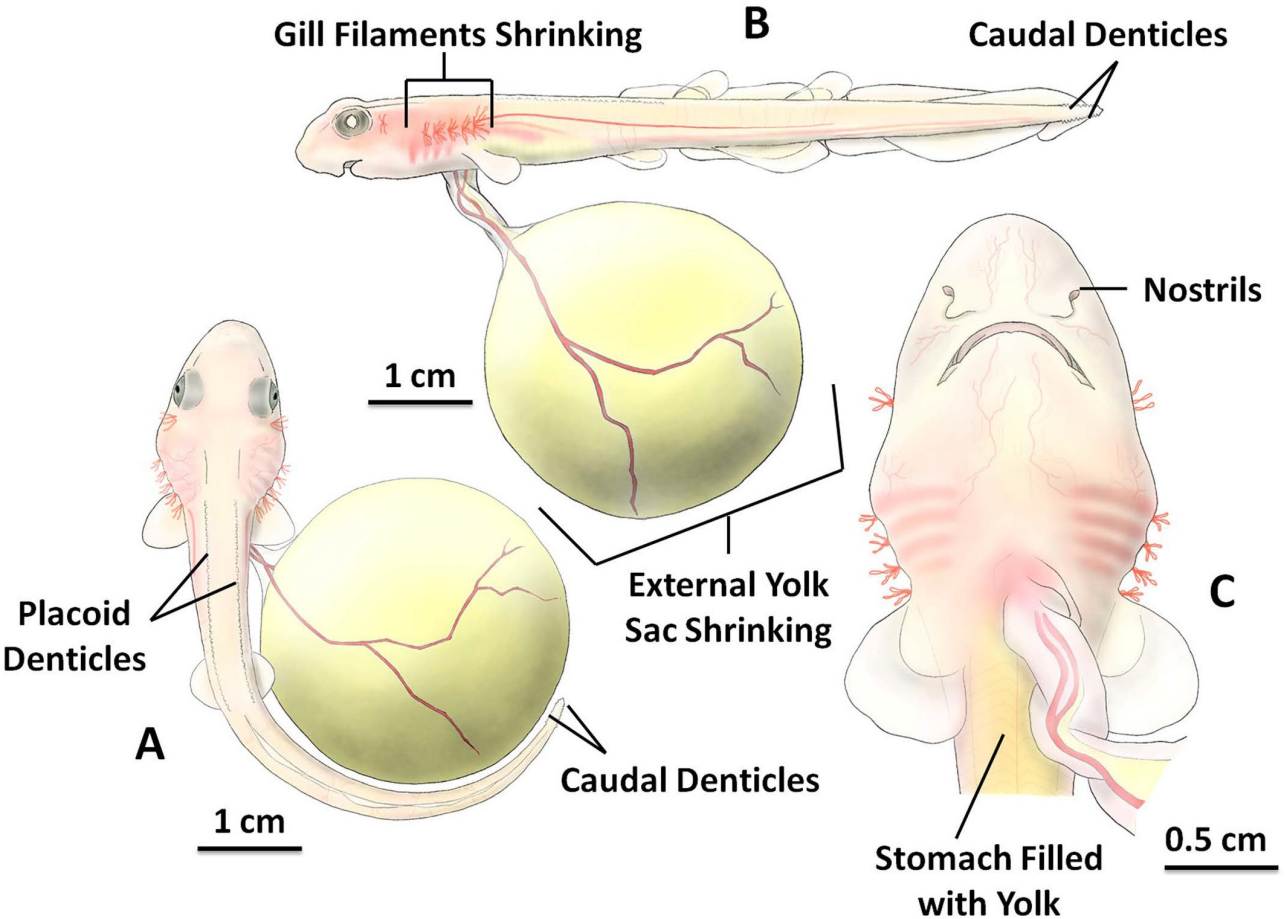
The inside of the *S*. *stellaris* egg case at stage 5. A: Position of placoid denticles and caudal denticles (dorsal view). B: Body shape began to resemble an adult shark and the external yolk sac shrank visible in size (lateral view). C: The magnified head and reduced gill filaments (ventral view). The key feature for stage 5 was nearly complete loss of external gill filaments. See S5 File for original photographs of Fig 5 illustrations.

The embryo usually reached stage 5 between 14 and 17 weeks after laying at 15 °C, with a mean age of 15.63 ± 1.19 weeks (n = 8). Stage 5 was characterized by the shrinking of the gill filaments, which could be observed non-invasively through the egg case; they were completely absent by the end of this stage (Fig 5). The total body length of stage 5 embryos ranged from 6.37 cm to 10.26 cm, with mean length of 7.57 ± 0.77 cm (n = 8). The embryo began to resemble an adult shark in body form and appearance. There were two rows of placoid denticles which can be seen in dorsal view (Fig 5A), and four rows of small caudal denticles located at the tail tip of the embryo. These small caudal denticles were uniformly positioned horizontally to the right, left, top and bottom of the tail tip (Fig 5A and 5B). More yolk mass was consumed to fuel growth and the external yolk sac was reduced to 38.79 ± 9.37% (n = 8) of its original size at stage 1 during stage 5. Due to this significant decrease, the reduction in external egg yolk size was now clearly visible. The embryo stomach was visibly filled with the ingested yolk mass, and the ventral stomach area changed to appear yellowish in color.

From the lateral view (Fig 5B), differences in the size of the fins were visible. Some of these fins were developing and growing larger, while others were decreasing in size, becoming completely absent by the end of stage 5. The gill filaments continued to shorten in length; one pair developed into spiracles and five pairs into gill openings toward the end of stage 5. The eyes had darker pigmentation at the center of each eye (Fig 5B) and the mouth was almost fully developed (Fig 5C).

From the ventral view of the embryo head (Fig 5C), it was possible to observe a pair of nostrils that had recently developed at the snout. More blood vessels developed around the snout during stage 5. The skin and flesh of the embryo grew thicker, and the whole embryo changed in color to pale pink, with more pigment developing at the later end of stage 5 as the skin thickened further. The heart beat was still visible near the yolk stalk, but the color intensity of the pumping blood appeared less intense than in the previous stage due to thickening of skin (Fig 5C).

### Stage 6

After 18 to 20 weeks (mean 19.25 ± 0.71 weeks, n = 8) post-laying at 15 °C, the embryo reached stage 6 which was characterized by the disappearance of fin-folds, which could be observed non-invasively through the egg case, and a significant increase in body size which ranged from 8.84 cm to 17.02 cm (mean length 10.84 ± 1.22 cm, n = 8). At this stage, the body was curled to maintain occupancy in the limited space inside the egg case. The size of the external yolk sac rapidly decreased during this stage and by the end of stage 6, the embryo had consumed 98.79 ± 1.05% (n = 8) of the original egg yolk volume measured at stage 1.

**Fig 6:**
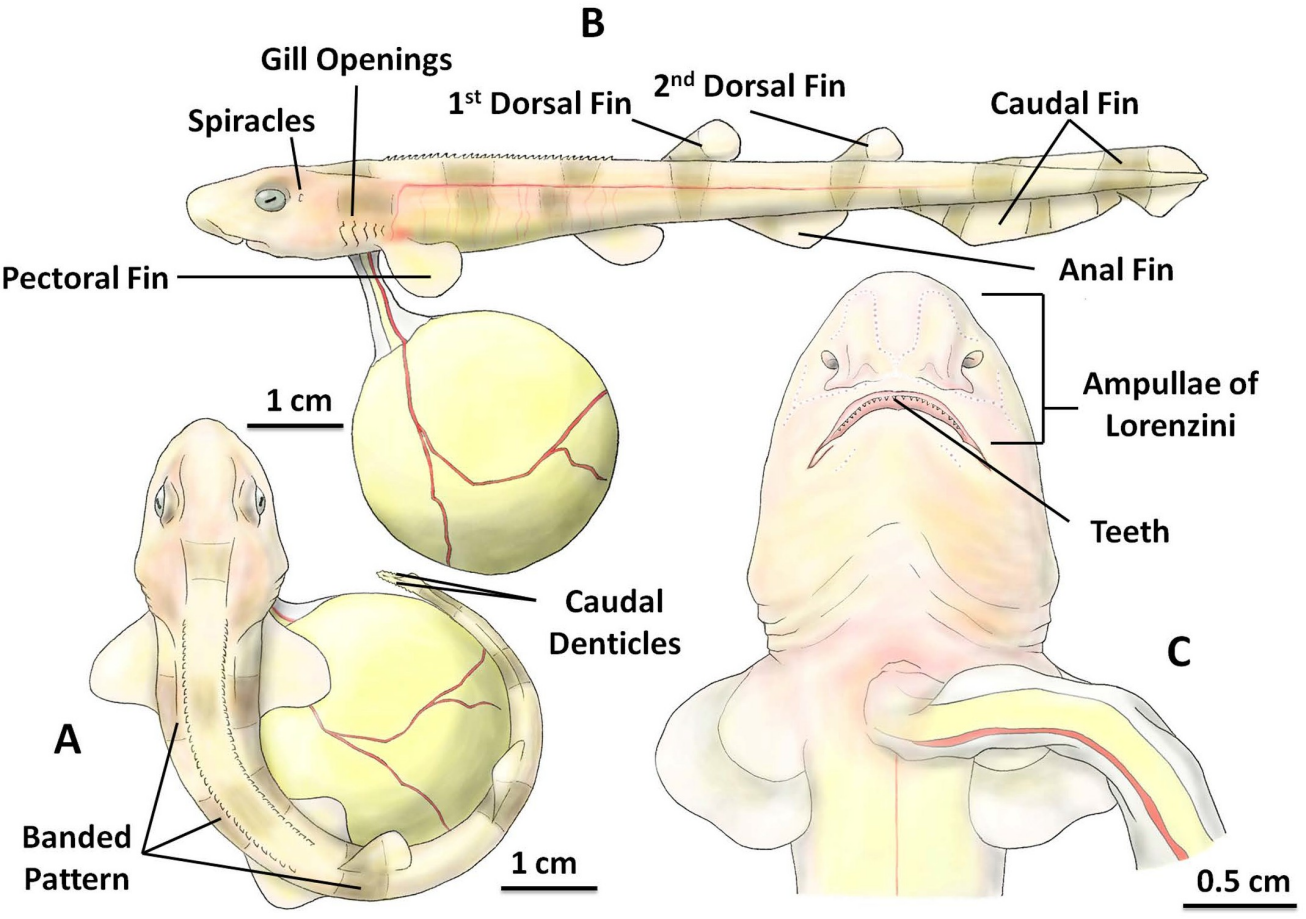
The inside of the *S*. *stellaris* egg case at stage 6. A: Position of placoid denticles, caudal denticles and banded patterns on the body (dorsal view). B: A pair of spiracles, five pairs of gill openings, and fins (lateral view). C: The magnified head (ventral view). The key feature for stage 6 was the loss of fin-folds. See S6 File for original photographs of Fig 6 illustrations.

At stage 6, the embryo had two rows of placoid denticles on top of its body, and two rows of small caudal denticles, which were located at the right and left side of the tail tip (Fig 6A). From the lateral view, clearly visible were two dorsal fins, a pair of pectoral fins, a pair of pelvic fins, an anal fin and a large dorsal and ventral caudal fin (Fig 6B). A pair of spiracles and five pairs of gill openings were fully developed as the gill filaments were completely shrunk (Fig 6B). More pigment had visibly developed on the skin by stage 6, resulting in banded patterns along the whole body that was yellowish peach with brownish grey to black bands, increasing in color intensity as the embryo developed further.

From the ventral view of the head (Fig 6C), the mouth was visible, with newly developed teeth inside the mouth opening. Furthermore, the ampullae of Lorenzini which had developed in earlier stages [13] were now visible with the naked eye (Fig 6C). The skin and flesh continued to grow thicker, the embryo became fully covered in small placoid scales, and the heart beat was no longer visible.

### Stage 7

**Fig 7:**
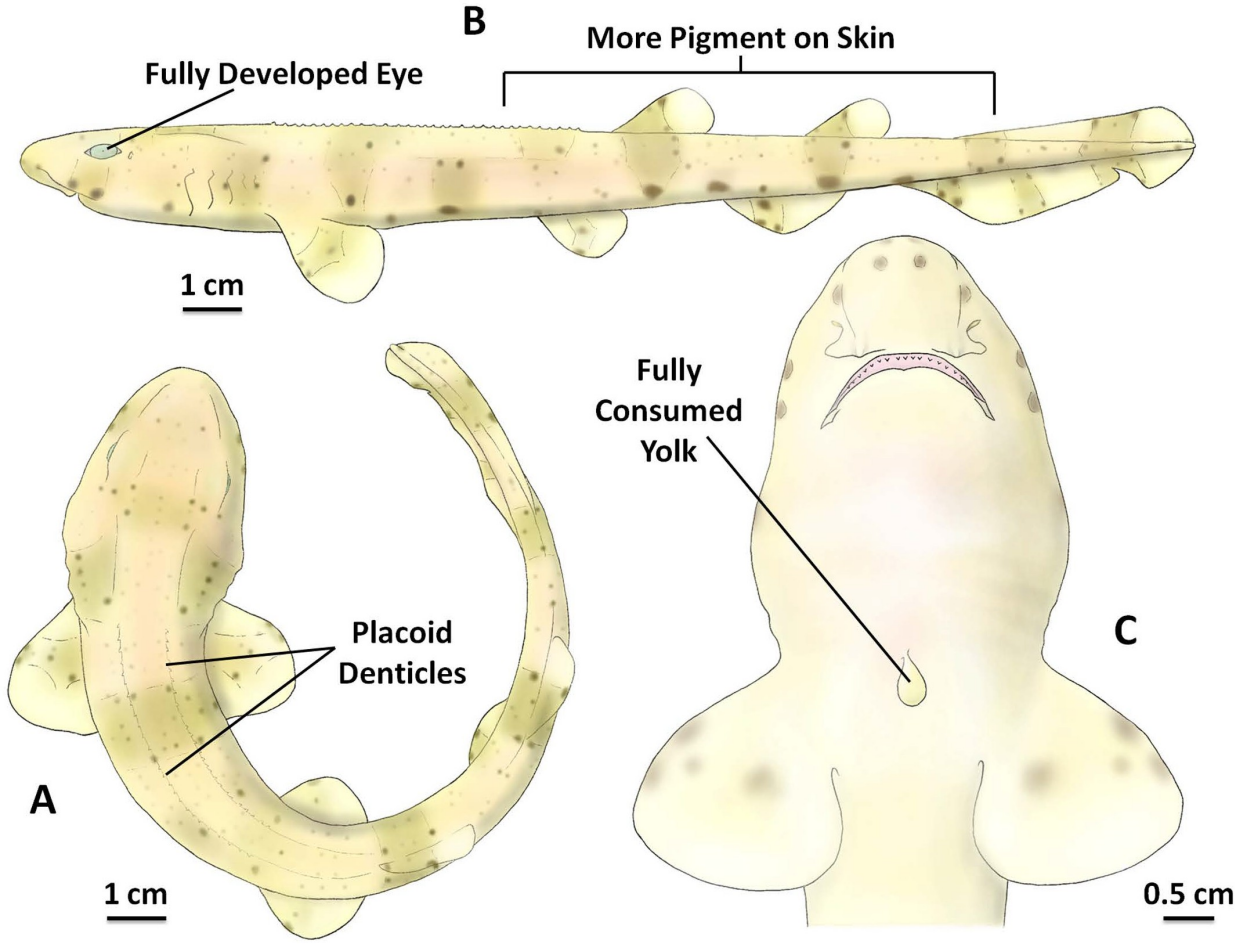
The inside of the *S*. *stellaris* egg case at stage 7. A: Position of placoid denticles and banded patterns on the body (dorsal view). B: Fully developed eye and greater pigmentation on skin (lateral view). C: The magnified head and fully consumed yolk (ventral view). The key feature for stage 7 embryo was a fully consumed external yolk. See S7 File for original photographs of Fig 7 illustrations.

Between 26 and 29 weeks (mean 27 ± 0.93 weeks, n = 8) post-lay at 15 °C the embryo reached stage 7 (Fig 7). This is the final stage of development before hatching out of the egg case. The total body length of stage 7 embryos ranged from 14.48 cm to 17.72 cm, with a mean length of 16.39 ± 0.93 cm (n = 8). Almost all of the yolk mass had been transferred from the external yolk sac into the internal yolk sac inside the embryo body. The yolk stalk and the external yolk sac membrane shrunk, which could be observed non-invasively through the egg case, and which was completely absent at the end of stage 7, leaving a smooth surface on the ventral side of the embryo (Fig 7C).

At stage 7, more pigmentation was visible, and the whole body color changed to yellowish brown, with brown banding and many dark spots (Fig 7A and 7B). As the small denticle scales developed, they gradually covered the whole body, and the embryo had a rougher textured skin. The eyes and other sensory organs were completely developed by this stage (Fig 7). At stage 7, the jelly inside of the egg case was fully degraded, and the flat end of the egg case membrane was weakened and could be easily opened fully.

**Fig 8:**
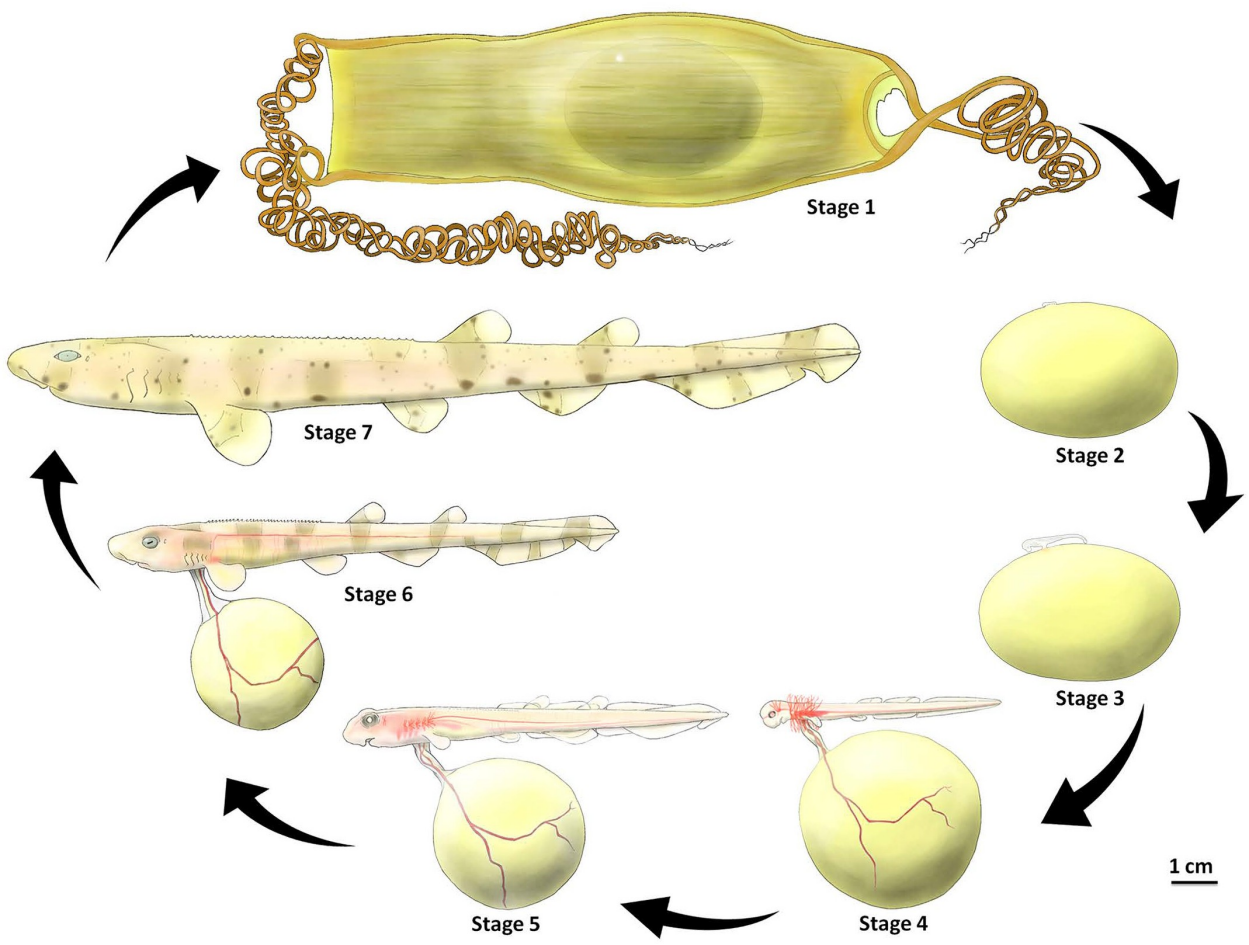
7 key developmental stages of *S*. *stellaris* inside the egg case. Stage 1 was characterized by no visible embryo. Stage 2 was identified as an early embryo formed on the surface of the egg yolk membrane. Stage 3 embryos had long tails and stage 4 embryos had externalized gill filaments. These filaments had shrunk by stage 5. In stage 6 the key feature was the loss of fin-folds, and in stage 7 the key feature was the fully consumed external yolk.

The developmental phases of *S*. *stellaris* in the 7 key stages presented to scale for size comparison in Fig 8. Furthermore, a summary of the 7 developmental stages is presented in Table 1, which includes the key features of each stage, the approximate age when the embryo reaches each stage (at 15 °C) and values for body length and yolk volume. Importantly, Table 1 also provides the same data for the closely related species, *S*. *canicula* which was the focus of Ballard’s [13] embryological scale. This allowed for a comparison between the 34 stages from Ballard *et al*. (1993) of *S*. *canicula* and the two species categorized using our 7 developmental stages here.

**Table 1:** Comparison of 7 developmental stages inside the egg case from *S*. *stellaris* and *S*. *canicula*.

7 developmental stages	34 stages of *S*. *canicula*, Ballard *et al*. 1993	Key features of each stage	Age *S*. *stellaris* (week)	Age *S*. *canicula* (week)	Body length *S*. *stellaris* (cm)	Body length *S*. *canicula* (cm)	Length range *S*. *stellaris* (cm)	Length range *S*. *canicula* (cm)	Yolk consumption *S*. *stellaris* (%)	Yolk consumption *S*. *canicula* (%)
**1**	4–16	No visible embryo	0	0	-	-	-	-	0%	0%
**2**	17–18	Embryo early formed	3	3	0.62 ± 0.2	0.47 ± 0.11	0.31–0.98	0.29–0.82	0%	0%
**3**	19–27	Long tail developed	4	4.25 ± 0.46	1.17 ± 0.16	1.1 ± 0.09	0.97–3.39	0.96–2.09	0%	0%
**4**	28–32	External gill filaments developed	8.13 ± 0.99	7 ± 0.53	3.64 ± 0.28	2.47 ± 0.21	3.24–8.32	2.18–5.75	< 28.05 ± 9.03%	< 22.21 ± 5.42%
**5**	33	External gill filaments shrunk	15.63 ± 1.19	14.13 ± 0.64	7.57 ± 0.77	6 ± 0.67	6.37–10.26	5.23–8.78	< 38.79 ± 9.37%	< 34.22 ± 7.23%
**6**	33	Fin-folds absent	19.25 ± 0.71	16.25 ± 0.46	10.84 ± 1.22	7.85 ± 0.91	8.84–17.02	6.44–10.63	< 98.79 ± 1.05%	< 98.35 ± 0.89%
**7**	34	Fully consumed external yolk	27 ± 0.93	21.38 ± 0.74	16.39 ± 0.93	10.3 ± 0.42	14.48–17.72	9.78–12.31	≈ 100%	100%

Key features are shown for each species developing inside the egg case. Data for both species (*S*. *stellaris* and *S*. *canicula*) are from this study (n = 8 for each species). The 34 stages of *Scyliorhinus canicula* (Ballard *et al*. 1993) was used to compare the detailed embryological staging to our non-invasive 7 developmental stages.

### Growth pattern and average daily body length gain (ADL)

**Fig 9:**
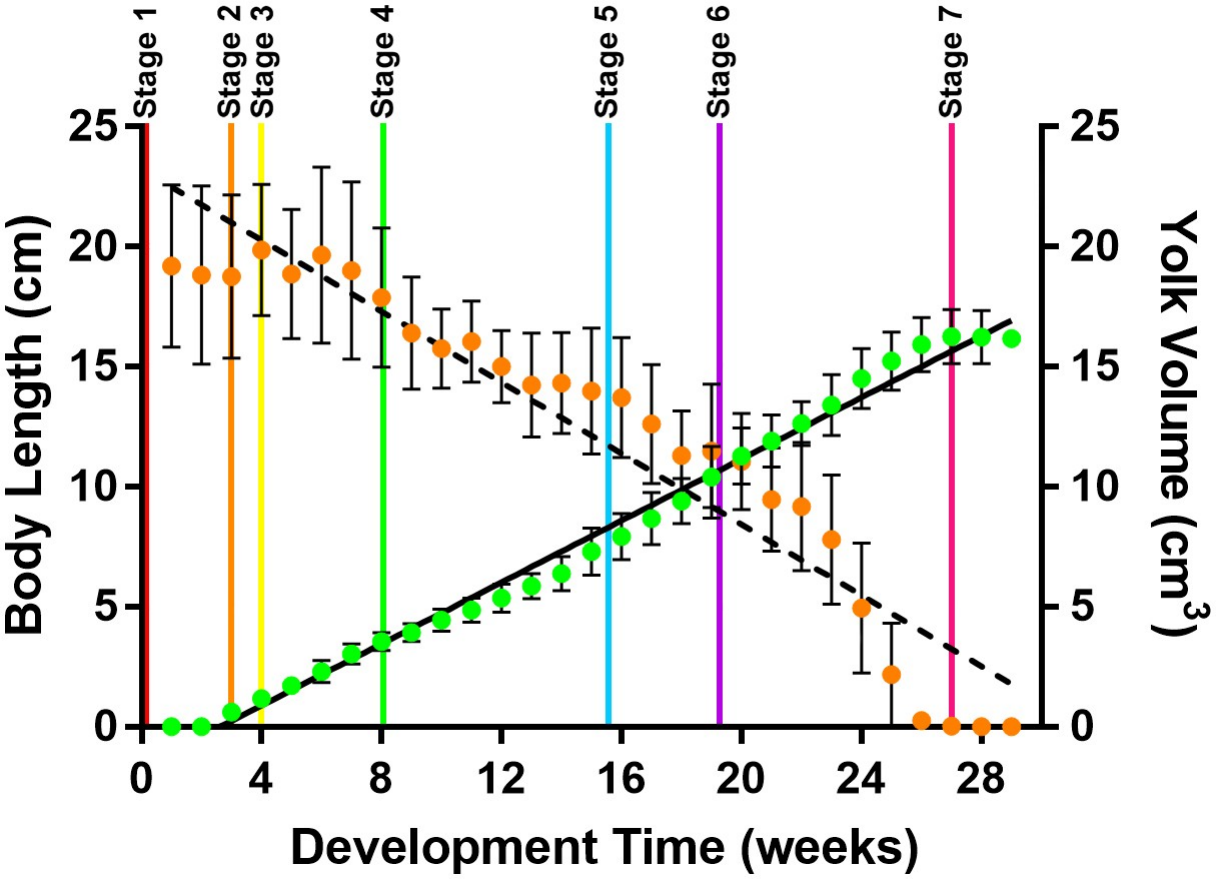
*S*. *stellaris* body length and external yolk sac volume across the 7 developmental stages. Total body length (cm, green, Y = 0.6416X − 1.674, R^2^ = 0.9898, r = 0.9949) and total external yolk sac volume (cm^3^, orange, Y = -0.7397X + 23.23, R^2^ = 0.9105, r = -0.9542) of *S*. *stellaris* embryo inside the egg case over development time (in weeks). Data are mean ± SD (n = 8). The beginning of each developmental stage is demarked with a vertical line.

**Fig 10:**
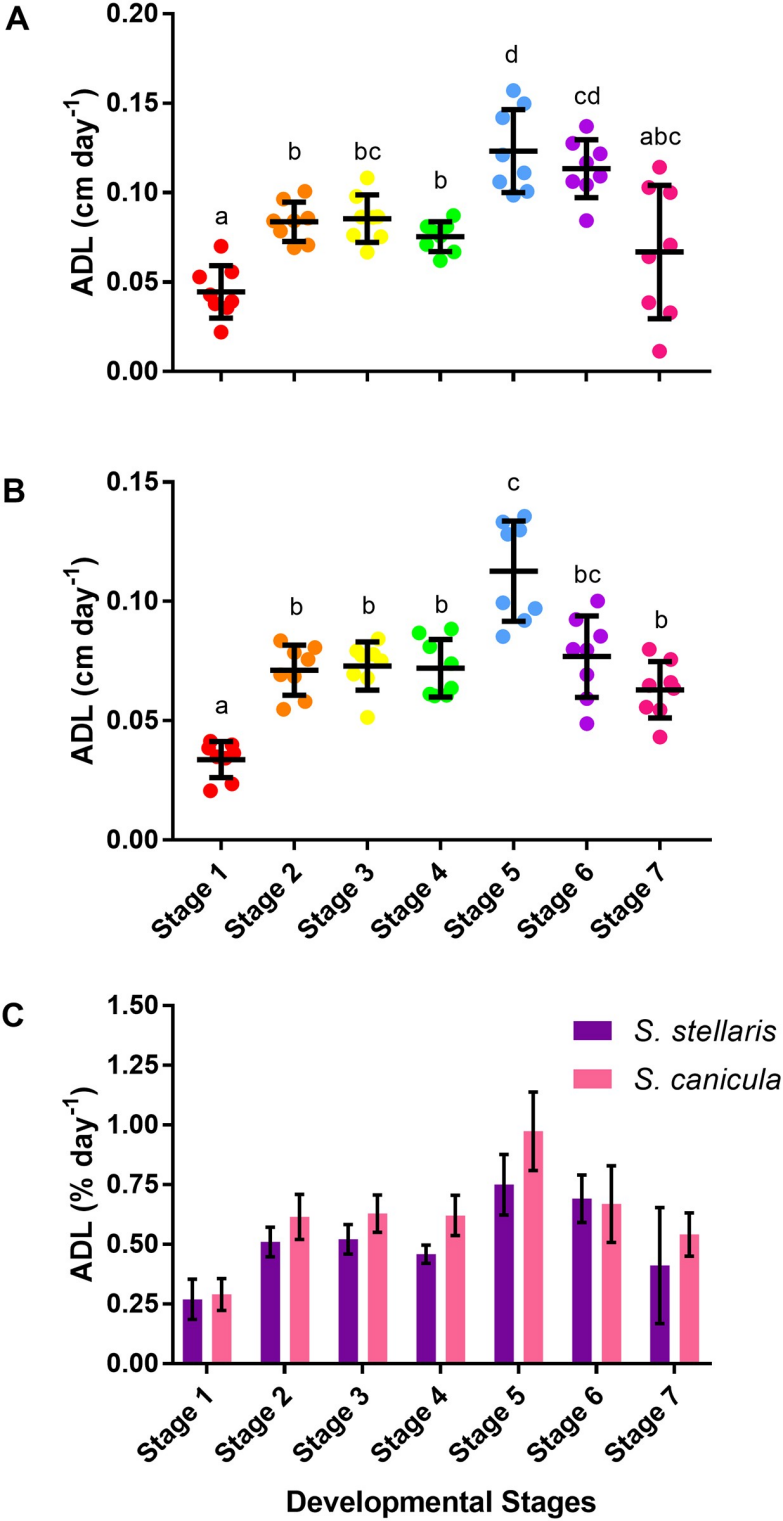
Average daily body length gain (ADL) during each of the 7 developmental stages. A: ADL (cm day^-1^) of *S*. *stellaris* embryos during each developmental stage. Lines show mean ± SD (n = 8), while points show measurements from each individual. Dissimilar letters indicate statistical differences between stages (RM one-way ANOVA, P < 0.05). B: ADL (cm day^-1^) of *S*. *canicula* embryos during each developmental stage. Lines show mean ± SD (n = 8), while points show measurements from each individual. Dissimilar letters indicate statistical differences between stages (RM one-way ANOVA, P < 0.05). C: Comparison of ADL (% day^-1^) between *S*. *stellaris* (purple) and *S*. *canicula* (pink) embryos at each developmental stage. Values represent mean ± SD (n = 8). There was no statistically significant difference between species at any stage (Mann-Whitney U test, P < 0.05).

*S*. *stellaris* growth inside the egg case was linear between the beginning of stage 2 and the end of stage 6 (Fig 9) however, the average daily body length gain (ADL) differed between developmental stages (Fig 10A). Slowest growth rate was observed in stage 1 whereas stage 5 had the highest growth rate (Fig 10A). A similar pattern was observed for ADL of *S*. *canicula* (Fig 10B). Indeed, no difference in ADL values (as % growth day^-1^) was found between species (Fig 10C).

### Yolk consumption rate

**Fig 11:**
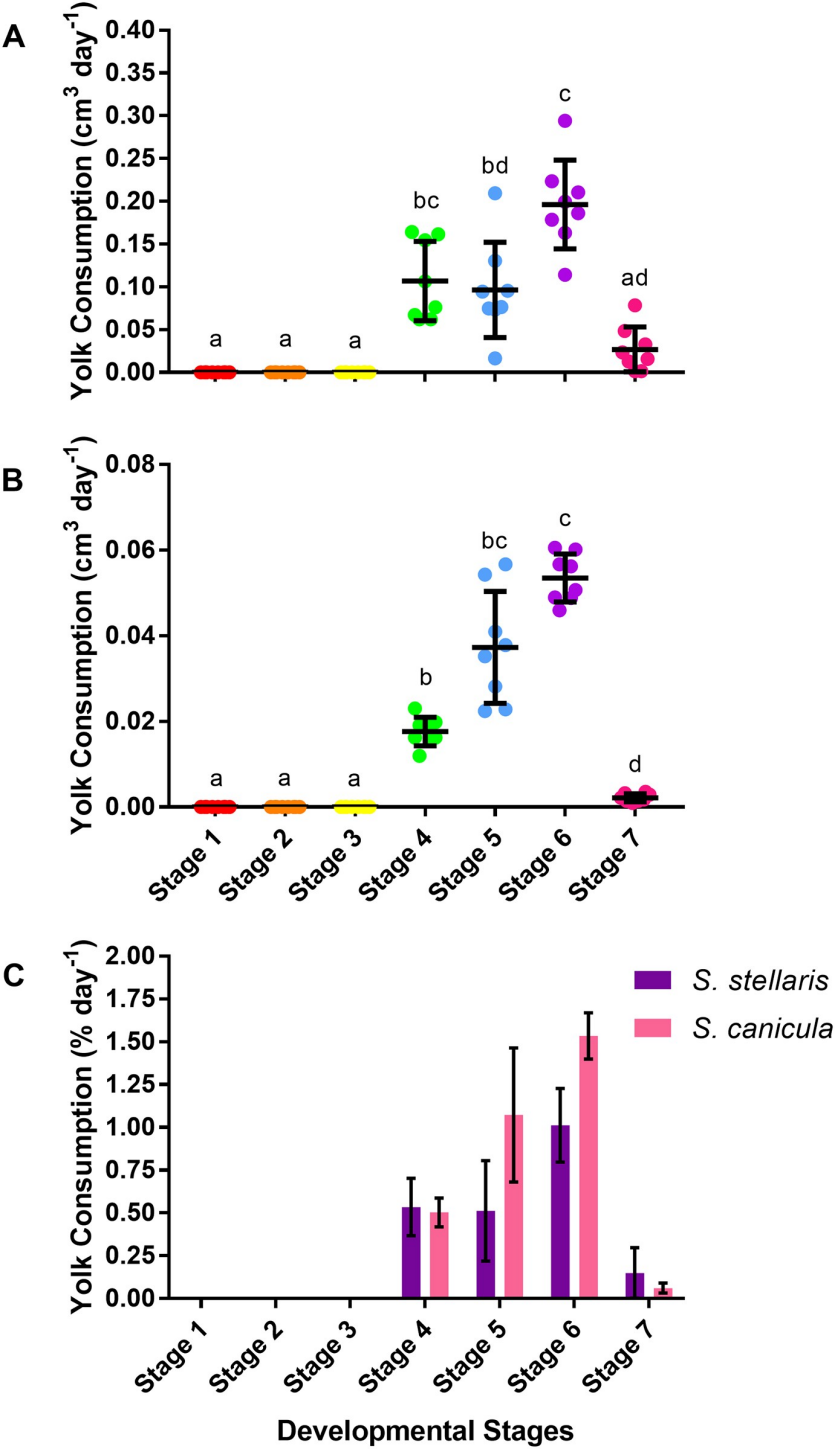
Yolk consumption rate during the 7 developmental stages. A: Yolk consumption rate (cm^3^ day^-1^) of *S*. *stellaris* embryos during the 7 developmental stages. Lines show mean ± SD (n = 8), while points show measurements from each individual. Dissimilar letters indicate statistical differences between stages (RM one-way ANOVA, P < 0.05). B: Yolk consumption rate (cm^3^ day^-1^) of *S*. *canicula* embryos during the 7 developmental stages. Lines show mean ± SD (n = 8), while points show measurements from each individual. Dissimilar letters indicate statistical differences between stages (RM one-way ANOVA, P < 0.05). C: Comparison of yolk consumption rate (% day^-1^) between *S*. *stellaris* (purple) and *S*. *canicula* (pink) embryos during 7 developmental stages. Values represent mean ± SD (n = 8). There was no statistically significant difference between species at any stage (Mann-Whitney U test, P < 0.05).

The decrease in the volume of the *S*. *stellaris* external yolk sac was visibly noticeable between stage 4 until stage 7 (Fig 9) with a variation in the rate of yolk consumption between stages, being the highest (0.2 ± 0.05 cm^3^ day^-1^) at stage 6 (Fig 11A). A similar pattern was observed for *S*. *canicula* (Fig 11B), and like growth rate (ADL), yolk consumption rate (as % day^-1^) at each stage did not differ between species (Fig 11C).

### Development time (embryo age and the duration of each developmental stage)

**Fig 12:**
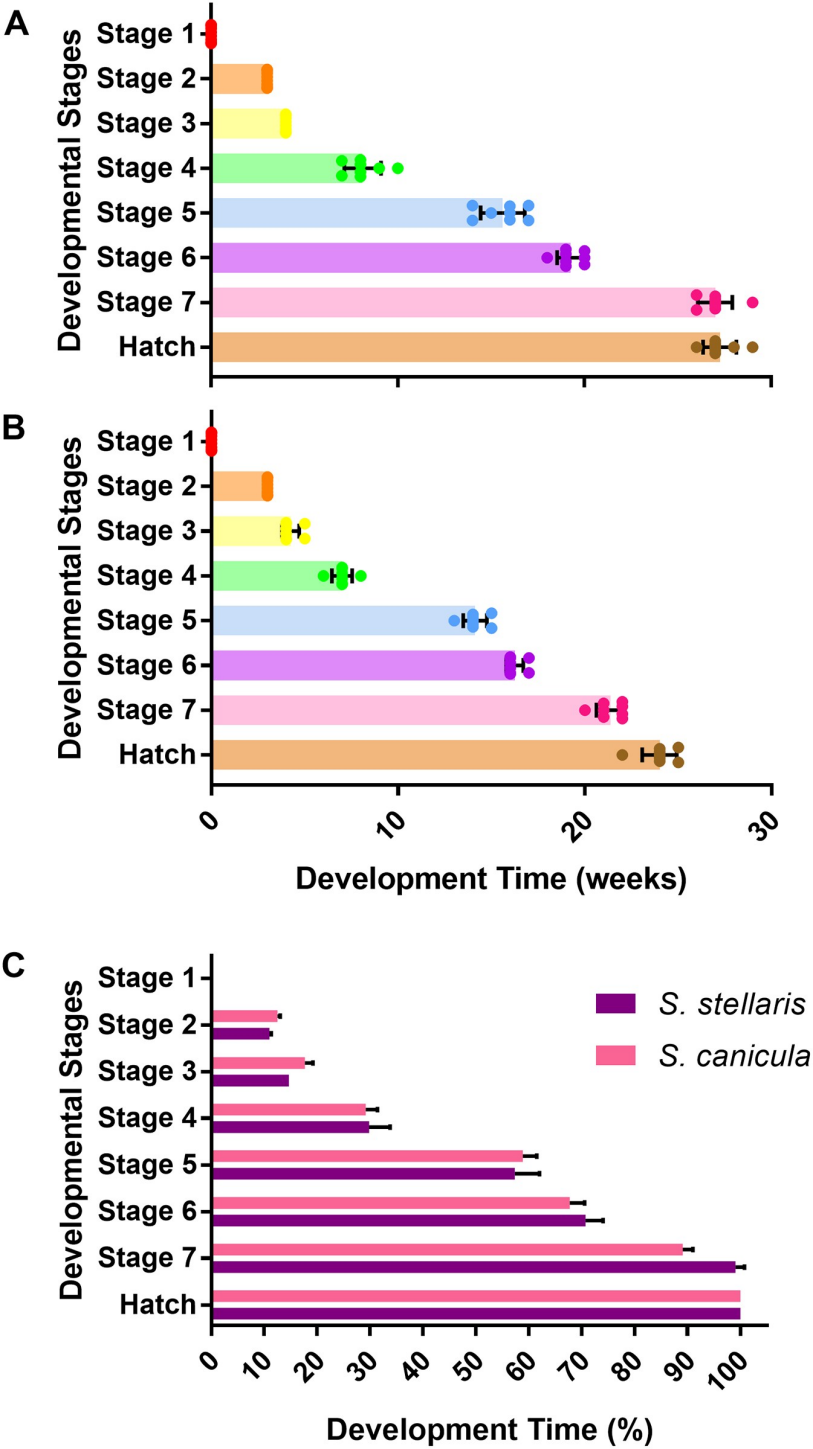
Development time (age) to reach 7 developmental stages and hatch. A: Development time (in weeks) for *S*. *stellaris* embryos to reach each developmental stage and hatch. Values represent mean ± SD (n = 8), whilst points show measurements from each individual. B: Development time (in weeks) for *S*. *canicula* embryos to reach each developmental stage and hatch. Values represent mean ± SD (n = 8), whilst points show measurements from each individual. C: Comparison of time spent in each developmental stage (as % of total developmental time) from lay to hatch for *S*. *stellaris* (purple) and *S*. *canicula* (pink). Values represent mean ± SD (n = 8). There was no statistically significant difference between species (Mann-Whitney U test, P < 0.05).

It took longer for *S*. *stellaris* (Fig 12A) to fully develop and hatch (27.25 ± 0.89 weeks) compared with *S*. *canicula* (24 ± 0.93 weeks) at the common rearing temperature of 15 °C (Fig 12B). However, the relative amount of time spent in each stage was consistent across species as expressed as the percentage of each species total developmental time spent in each stage (Fig 12C).

**Fig 13:**
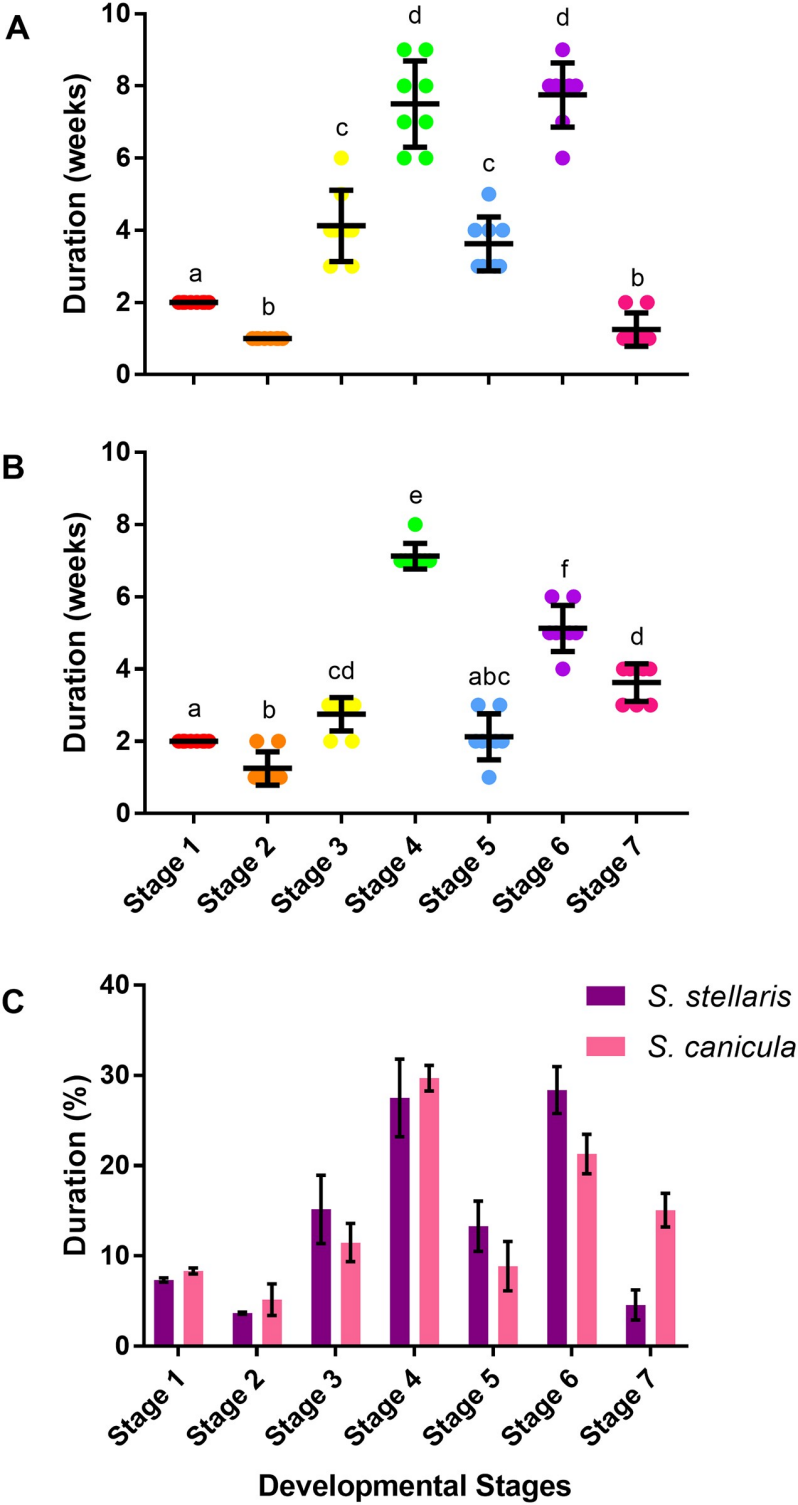
Duration of development time of the 7 developmental stages. A: Duration of development time (in weeks) spent in each of the 7 developmental stages for *S*. *stellaris* embryos. Lines show mean ± SD (n = 8), while points show measurements from each individual. Dissimilar letters indicate statistical differences between stages (RM one-way ANOVA, P < 0.05). B: Duration of development time (in weeks) spent in each of the 7 developmental stages for *S*. *canicula* embryos. Lines show mean ± SD (n = 8), while points show measurements from each individual. Dissimilar letters indicate statistical differences between stages (RM one-way ANOVA, P < 0.05). C: Comparison of duration of development time (as % of total time) between *S*. *stellaris* (purple) and *S*. *canicula* (pink) embryos at 7 developmental stages. Values represent mean ± SD (n = 8). There was no statistically significant difference between species (Mann-Whitney U test, P < 0.05).

The amount of time (in weeks) that *S*. *stellaris* spent at each developmental stage is shown in Fig 13A. Embryos spent the least amount of time in stage 2 (a week) and the longest developmental period in stage 6 (7.75 ± 0.89 weeks). A similar pattern was observed for *S*. *canicula* (Fig 13B), which each species spending a similar proportion of their development in a given stage (Fig 13C).

### Hatching process

**Fig 14:**
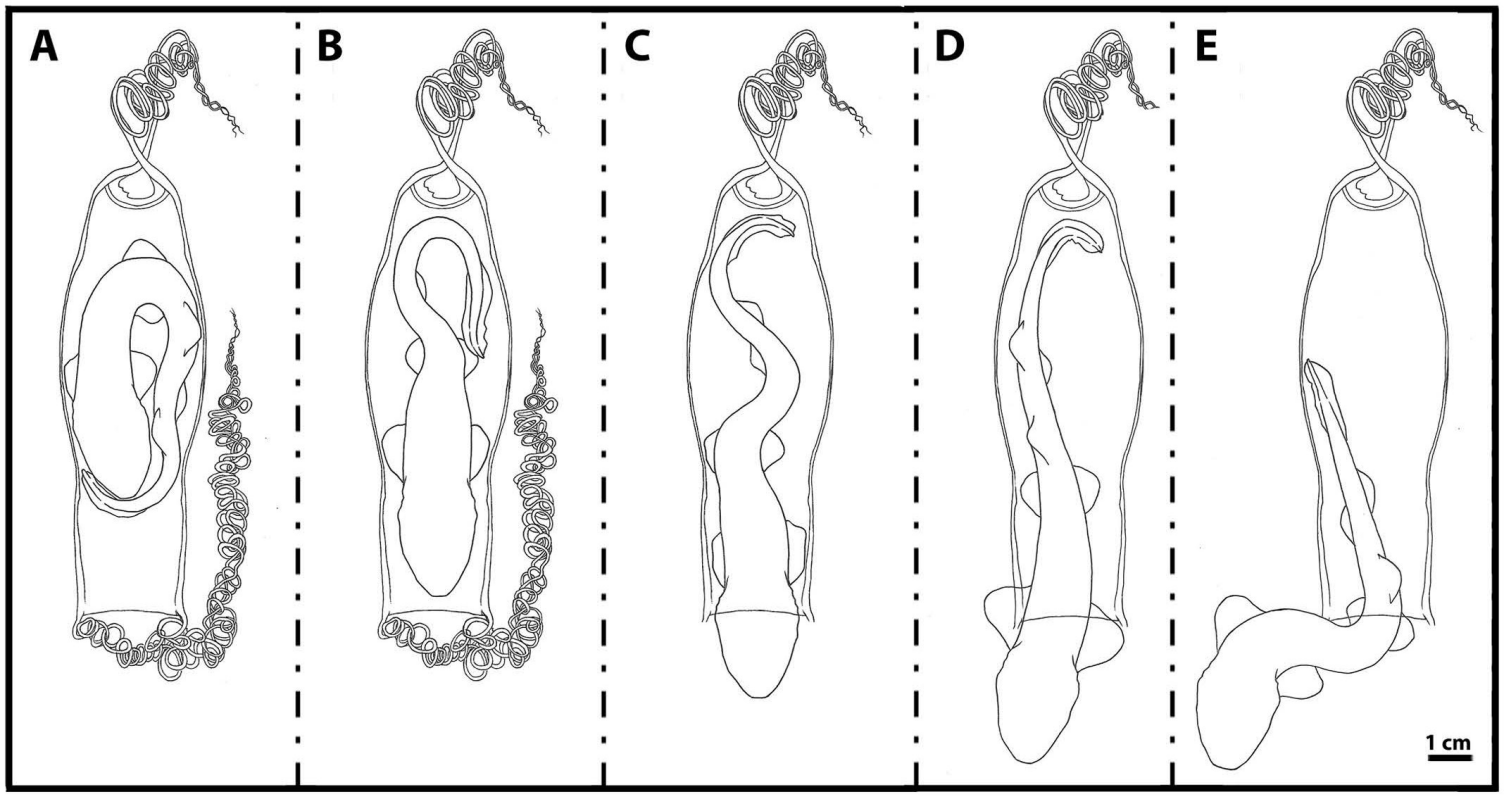
The hatching process. A: Embryo with curled body inside the egg case. B: Embryo facing the flat end of the egg case. C: Embryo tail pushed against arched wall of the egg case. D: Embryo pushed its body through the flat end of the egg case. E: Embryo swam out of the egg case.

The embryo positioned itself, immobilized inside the egg case, with half of its body curled to fit the limited space (Fig 14A). Prior to hatching, it rotated such that the head was pointed toward the flat end opening of the egg case (Fig 14B). Next, the tail struggled and pushed against the rigid, arched wall of the egg case, which forced the head through the flat end opening of the egg case (Fig 14C).

The embryo slowly and gradually pushed from the inside of the egg case, struggling until most of the abdomen was successfully out of the egg case membrane (Fig 14D). Once the abdomen had emerged from the egg case, the tip of the tail touched the surface of the arched wall end of the egg case to push the body forward enough that the embryo could then swim out through the egg case opening (Fig 14E).

The duration of the hatching process varied greatly between the observed individuals, ranging from a few minutes to more than one hour to completely escape the egg case. At hatch, determining the sex of *S*. *stellaris* hatchlings was possible because the male hatchlings had a pair of short claspers between the pelvic fins, whereas the females did not.

### Effect of air exposure on *S*. *stellaris* egg cases with jelly and without jelly

**Fig 15:**
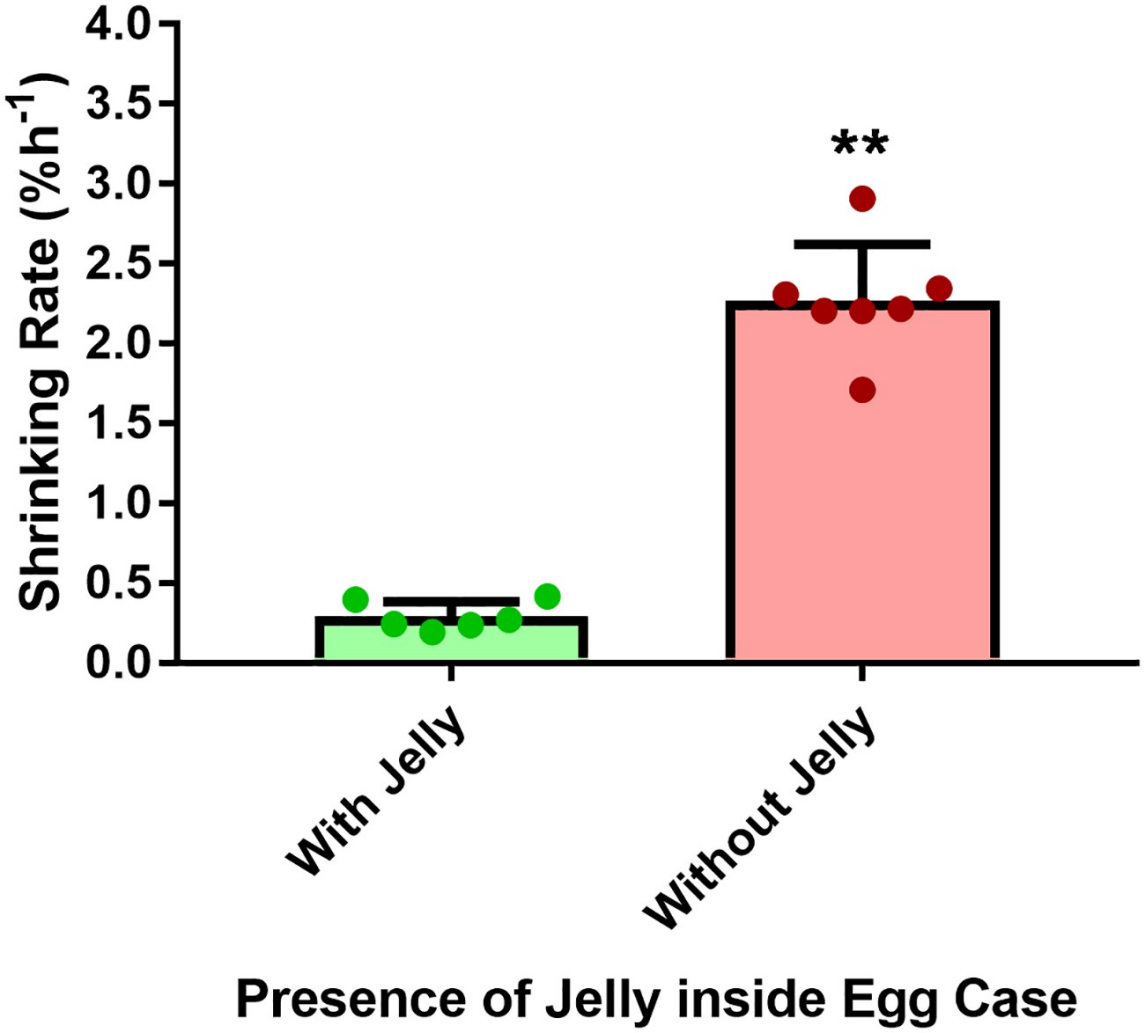
Shrinking rate of *S*. *stellaris* egg cases (% in area h^-1^) with and without jelly. Values represent mean ± SD, while points show individual measurements. Asterisk indicates statistically significant effect of jelly on the rate of egg case shrinking (Mann-Whitney U test, P < 0.05).

Unfertilized *S*. *stellaris* egg cases which were filled with jelly were able to maintain their shape and size for 1–2 days when aerially exposed. In contrast, the egg cases without jelly, which were discarded from hatched individuals, retained their shape and size for only one hour before starting to shrink. Thus, *S*. *stellaris* egg cases without jelly had much faster dehydration rate (2.27 ± 0.35% day^-1^) compared to the egg cases with jelly (0.29 ± 0.09% day^-1^) (Fig 15) suggesting that the jelly protects air exposed embryos by slowing dehydration/shrinking rate by ~7.8 times.

## Discussion

Here we produced the first oviparous elasmobranch embryonic developmental scale that can be applied non-invasively, without specialized equipment, to help approximate embryo age, lay time and hatch time, whilst in the field. The 7 developmental stages were identified based on the greater spotted catshark, *Scyliorhinus stellaris*, and validated against the closely related elasmobranch species, *S*. *canicula* (the same species used in Ballard *et al*. 1993 embryonic elasmobranch developmental scale). More distantly related oviparous elasmobranch species, such as the tropical, brownbanded bamboo shark [26], and the batoid elasmobranch species, the clearnose skate [14] show similar key features during their embryonic development inside the egg cases, as presented here in our 7 key stages scale. Thus, we suggest it can be extended to other oviparous shark species with only slight modification.

The 7 specific developmental stages were chosen because they are recognizable by the naked eye, can be observed non-invasively (without the needing to remove embryo from the egg case), and also are ecologically and physiologically important in allowing the elasmobranch inside the egg case to survive in intertidal zone. Stage 1 was chosen as it did not have a visible embryo, while stage 2 had an embryo at the earliest stage of development. Stage 3 embryos had developed a tail and the embryo movement was nearly continuous. During stage 4, long gill filaments developed and respiratory process beyond cutaneous diffusion occurred. At stage 5, gill filaments shrunk and major changes in shape and body morphology occurred as the animal grew. The stage 6 embryo was characterized by the loss of fin-folds, and an increase in yolk mass transfer from the external yolk sac. The stage 7 embryo was fully developed, with a completely consumed external yolk and ready to hatch. We also were able to determine some unique features of the egg case and the protective jelly encasing the developing embryo that may contribute to survival in the harsh intertidal zone environment.

### Early embryogenesis

Although not visible with the naked eye, after fertilization, embryos at stage 1 undergo several important development processes. The rapid division of cells (cleavage) from one large cell (the zygote) into many smaller cells (called blastomeres) produce a three-dimensional cell mass cluster (the morula), which later develops into a hollow spherical cell mass with a fluid-filled cavity, the blastocoel (the blastula). During gastrulation (embryo during this process called a gastrula), the epiboly of blastoderm occurred, where the cells migrate to form germ layers (the outer layer of ectoderm, the inner layer of endoderm, and the middle layer between these two, called mesoderm), which will allow the later formation of tissues and organs as the embryo develops [13]. These changes are difficult to be observed with the naked eye, thus during stage 1, the embryo (the blastodisc) can only be seen as a small spot located on the surface of the yolk membrane as shown in Fig 1. As gastrulation continues, more cells migrate into the interior of the embryo forming the primitive streak at the blastoderm’s midline [13]. The thickening of primitive streak gives rise to the elevation of the embryo on the surface of the yolk in stage 2 (Fig 2).

### Egg case structure and function

The shape and morphology of elasmobranch egg cases are unique and diverse. Different oviparous elasmobranch species have egg cases with different shapes and morphological features, thus the elasmobranch egg case can be used for species identification [2,3]. There are many color variations due to the catechol oxidation reaction [27]. We found different color variations of the same elasmobranch egg case species (for *S*. *stellaris* and *S*. *canicula*), hence egg case color cannot be used for species identification.

Features of the *S*. *stellaris* egg case increase embryonic survival in the extreme habitats where they are laid. The tough egg case membrane acts as the primary protection for the developing embryo against marine predators and parasites [22–24] and provides protection from seawater exposure during early development (stages 1–4) [13]. The egg cases are also used as camouflage as the egg case itself resembles blades of kelp and the tendrils are similar in form to kelp holdfasts [2,21]. The tendrils, which we found to be easily tangled, are sturdy, flexible, and have a spring-like structure which helps in securing egg cases underwater against the strong currents of the intertidal zone [16].

The *S*. *stellaris* egg case shape also appears to help the hatchling escape during hatch (Fig 14). The curvature of the arched end of the egg case may provide increased surface for the tail push against and force the embryo through the small, thin egg case opening. Similar interaction between the hatching process and the egg case has also been observed in the clearnose skate, *Raja eglanteria* [28], whose egg case has a similar morphology.

### Jelly as protection

The embryonic jelly provides secondary protection to the developing embryo with the egg case membrane being the primary. The jelly acts as a shock absorber, protecting the embryo from strong waves in the intertidal zone [29,30]. The tougher outer layers provide support and sealing of the egg case membrane preventing seawater entry, while the centre of the jelly is softer and is thought to providing cushioning from wave action. Indeed, these layers are known to differ in carbohydrate composition [29].

We also found that egg cases containing jelly maintained their shape and size nearly 8 times longer than those without jelly during air exposure (Fig 15). This suggests that the jelly prevents the egg case membrane from rapidly drying and shrinking when washed up on a beach or in shallow rockpools. By slowing down the dehydration rate of the egg case, the jelly protects the embryo during short term air exposure.

### Opening of seawater slits

During the earliest developmental stages (stages 1–3), the egg yolk membrane of *S*. *stellaris* is thin and fragile. As the epiboly of blastoderm occurred during gastrulation, the formation of germ layers (ectoderm and mesendoderm) spreading around the yolk [13] resulting the external yolk sac became thicker and tougher during stage 4, before the seawater slits open at the end of stage 4. This fragility of the yolk membrane during the early developmental stages has been reported in other elasmobranch species [13,30–32]. Ballard *et al*. (1993) suggested that embryos directly exposed to seawater during early developmental stages could die due to bacterial exposure from seawater [13].

At stage 4, (equivalent to stage 28 from *Scyliorhinus canicula*, Ballard *et al*. 1993), enzymes from the hatching gland gradually digest the jelly, starting with the soft central jelly and, then gradually working towards the edge of the egg case with its tougher texture, near to the seawater slits [13]. Once most of the tough jelly near the edge of the egg case has been digested, the mucous plugs degrade and the seawater slits open exposing the embryo to seawater. Similar observations had also been reported in the Port Jackson shark, *Heterodontus portusjacksoni* egg case [30].

In this study, by the end of stage 4, all four seawater slits were fully opened and the seawater could freely enter into the egg case. This confirms that *S*. *stellaris* is tolerant to seawater exposure by this stage, similar to previous descriptions for *S*. *canicula* and *H*. *portusjacksoni* [13,30]. Some studies initiated earlier seawater exposure for other elasmobranch species [13], however when replicated in this study, *S*. *stellaris* survived for only a few weeks or months, with none surviving beyond 3 months if exposed to seawater before stage 4. This suggests that for this species, stage 4 is the earliest developmental stage that is suitable for seawater exposure.

### Gills and respiration

During early development (stages 1–3), while the egg case membrane is fully sealed, the jelly provides adequate medium for gas exchange across the body wall [16,33–35]. At the end of stage 3, capillaries begin to develop on the surface of the large external yolk sac providing a surface area larger than the body wall for gaseous exchange [34,36].

During stage 4, metabolic requirements begin to increase and the inside of the egg case can become hypoxic [16]. The internal gills extend out from the gill openings, becoming the thin walled external gill filaments [16,37], which together with the degradation of the jelly provide increased access to oxygen. *S*. *stellaris* and *S*. *canicula* embryos spend many weeks in stage 4 (Fig 13), where changes in gill morphology allow them to cope with the growing hypoxia inside the egg case and prepare them for the opening of the seawater slits at the end of this stage.

After the slits open and the inside of the egg case returns to normoxia, the length of external gill filaments gradually shrink during stage 5 and the external gill buds have completely disappeared at the beginning of stage 6. By this stage, buccal pumping of the embryo gill openings was clearly observable. The internal gills are completely formed in stage 6 and the gills are fully functional for respiration.

### Fin and body morphology

Fin morphology changed during embryonic development with fins emerging and then regressing. During stage 4, the embryo was equipped with a pair of pectoral fins, a pair of pelvic fins and large, long dorsal and ventral fin-folds along the tail. As the embryo grew in size, the added surface area provided by the fin-folds may have improve fluid mixing of enzyme and jelly inside the egg case [13,16]. Lastly, the fin-folds may be important in adding surface area for ventilation of seawater through all four fully opened seawater slits to supply fresh oxygenated seawater to the developing embryo.

In stage 5, the embryo has the greatest ADL (Fig 10) and thus body and tail movements alone may facilitate adequate ventilation inside the egg case, thus the excessive fin-folds are reduced dramatically during this time. Some of these fin-folds extended outwards becoming fins, while most of the fin-folds gradually shrunk and completely disappeared by stage 6. At stage 6, the embryo was fully equipped with two dorsal fins, a pair of pectoral fins, a pair of pelvic fins, an anal fin as well as a large dorsal and ventral caudal fin which are typical of the adult shark. The large caudal fin will be used for forward propulsion [38], the dorsal fins function as stabilizers [39], and the flexible pectoral and pelvic fins help in steering while swimming and in holding on to the substrate during resting [40,41].

### Yolk consumption during development

There was no visible yolk consumption by the embryo during the early developmental stages (stages 1–3) for both *S*. *stellaris* and *S*. *canicula* (Fig 11). The external yolk membrane was thin and not fully developed, making the external yolk sac an ellipsoid [13]. The development of blood capillaries and thickening of external yolk sac membrane resulted in an apparent increase egg yolk volume during early development as the shape changed from ellipsoid to spheroid (Figs 8 and 9). By stage 3, the yolk stalk began to develop as the embryo grew larger and more energy was required to support development.

The yolk stalk was completely developed by stage 4, and yolk mass was transferred from the external yolk sac into the embryo’s internal yolk sac and digested, however changes in yolk volume were not appreciable visually at this stage [13,42,43]. This has been previously described for *S*. *canicula* [13]; the yolk mass starts to be transferred from external yolk sac to internal yolk sac at stage 31 in Ballard *et al*. (1993), which is equivalent to our stage 4. By stage 5, the decreased size of the external yolk sac was noticeable by the naked eye and yolk shrank most rapidly during stage 6 (Fig 11). At stage 7, the yolk mass had been fully transferred from the external yolk sac into the embryo for growth, though some of the yolk was transferred into the internal yolk sac inside the body of embryo [13,42–44]. Once hatched, the embryo is able to utilize the yolk mass stored inside the internal yolk sac until it is successful in hunting prey [43].

## Conclusions

7 key stages of embryonic development were identified for *S*. *stellaris* that can be identified by the naked eye in living elasmobranch embryos. As elasmobranchs are sentinels for ecosystem health, and because embryonic life stages are particularly vulnerable, being able to monitor key developmental stages non-invasively is vital for eco-physiology and conservation studies. Our developmental stages are provided alongside the gold standard embryological key developed by Ballard *et al*. (1993), to provide in depth information on features that cannot be seen non-invasively. Our 7 key developmental stages scale can thus be applied non-invasively to living elasmobranch egg cases, without specialized equipment or additional fixation procedures. It is designed to be user-friendly to non-embryological researchers and the general public for development staging in the field. Thus we hope that this will allow a wider audience to contribute towards elasmobranch conservation efforts and research.

## Supporting information

S1 File Original photographs of Fig 1 illustration. External features of the *S*. *stellaris* egg case at stage 1.
(PDF)

S2 File Original photographs of Fig 2 illustrations. The inside of the *S*. *stellaris* egg case at stage 2.
(PDF)

S3 File Original photographs of Fig 3 illustrations. The inside of the *S*. *stellaris* egg case at stage 3.
(PDF)

S4 File Original photographs of Fig 4 illustrations. The inside of the *S*. *stellaris* egg case at stage 4.
(PDF)

S5 File Original photographs of Fig 5 illustrations. The inside of the *S*. *stellaris* egg case at stage 5.
(PDF)

S6 File Original photographs of Fig 6 illustrations. The inside of the *S*. *stellaris* egg case at stage 6.
(PDF)

S7 File Original photographs of Fig 7 illustrations. The inside of the *S*. *stellaris* egg case at stage 7.
(PDF)
