# Supplementary material for: Oviparous elasmobranch development inside the egg case in 7 key stages
Source: PLoS One. 2018 Nov 6;13(11):e0206984. doi: 10.1371/journal.pone.0206984 (PMC6219803; doi:10.1371/journal.pone.0206984)

Original photographs of Fig 1 illustration. External features of the *S. stellaris* egg case at developmental stage 1.

Fig 1

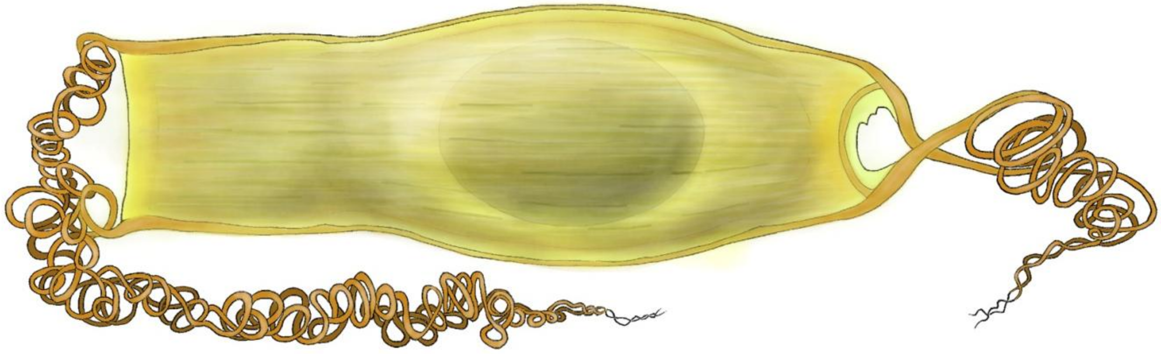

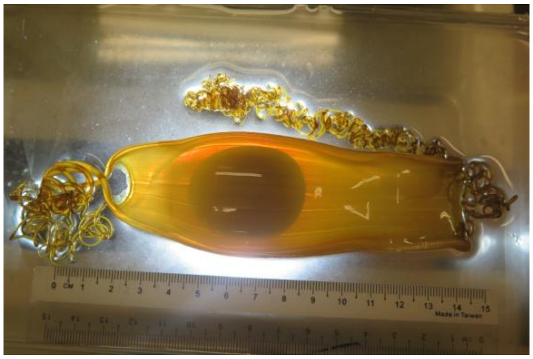

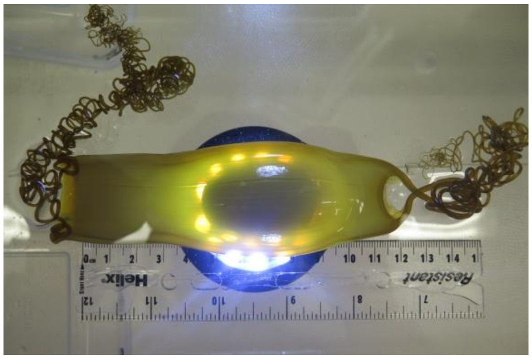

Supplement: S1 File — External features of the S. stellaris egg case at stage 1. (PDF) [file pone.0206984.s001.pdf]
