# Supplementary material for: Oviparous elasmobranch development inside the egg case in 7 key stages
Source: PLoS One. 2018 Nov 6;13(11):e0206984. doi: 10.1371/journal.pone.0206984 (PMC6219803; doi:10.1371/journal.pone.0206984)

Original photographs of Fig 6 illustrations. The inside of the *S. stellaris* egg case at developmental stage 6 with the egg yolk mass and the associated embryo.

Fig 6A

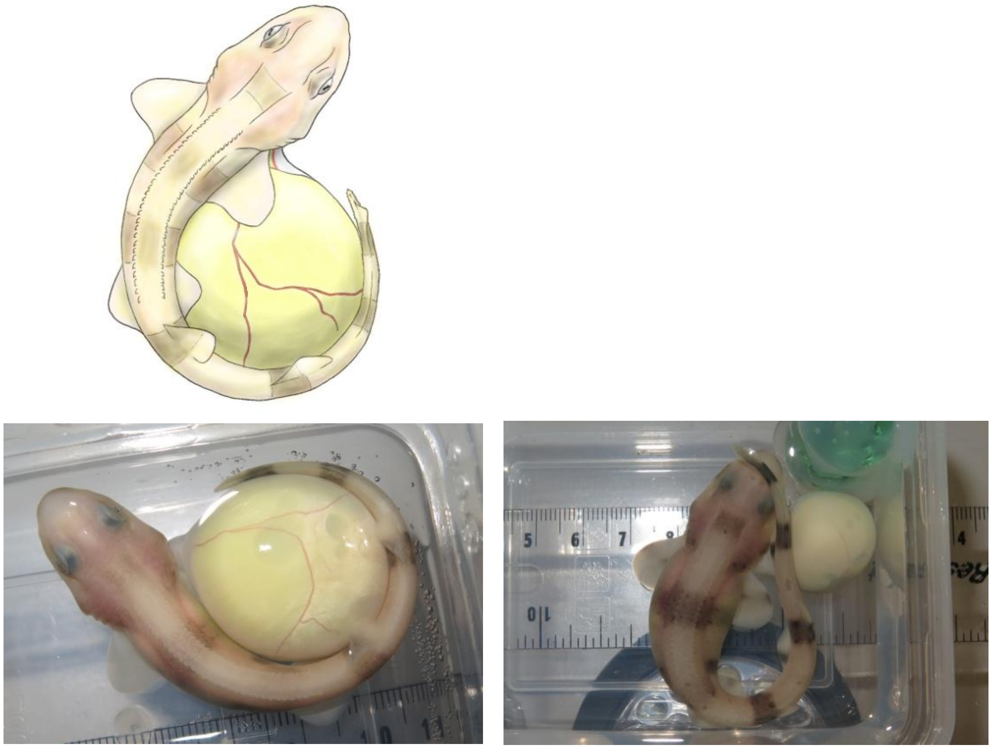

Fig 6B

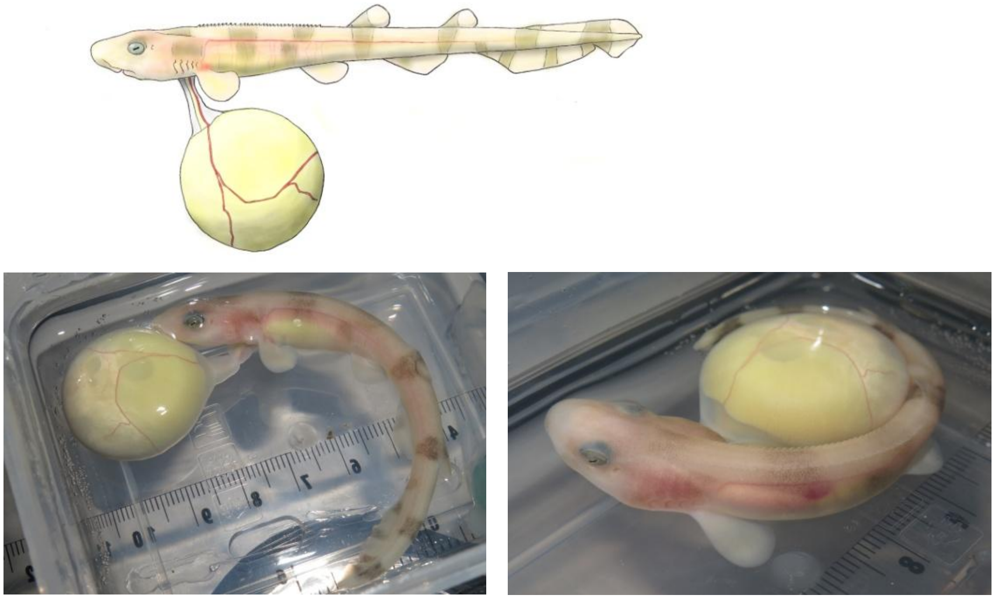

Fig 6C

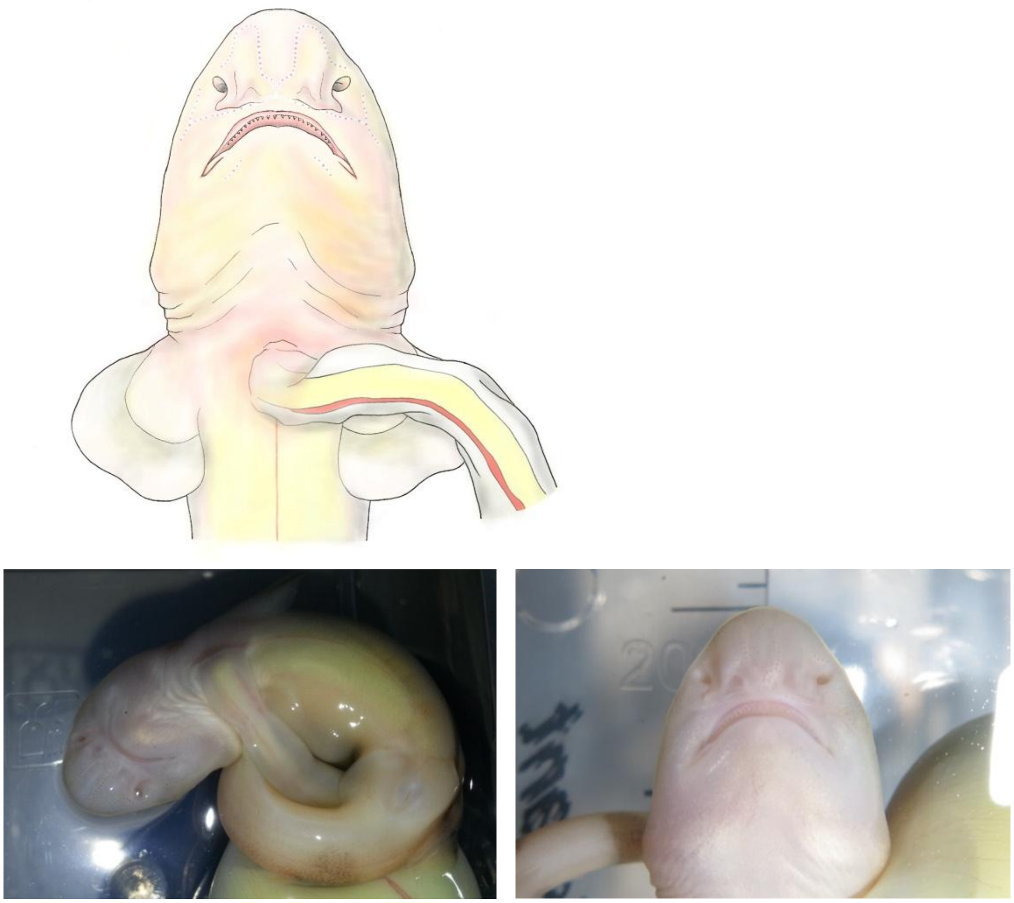

Supplement: S6 File — The inside of the S. stellaris egg case at stage 6. (PDF) [file pone.0206984.s006.pdf]
