# Supplementary material for: Oviparous elasmobranch development inside the egg case in 7 key stages
Source: PLoS One. 2018 Nov 6;13(11):e0206984. doi: 10.1371/journal.pone.0206984 (PMC6219803; doi:10.1371/journal.pone.0206984)

Original photographs of Fig 7 illustrations. The *S. stellaris* embryo inside of the egg case at the end of developmental stage 7 with the egg yolk mass completely absorbed.

Fig 7A

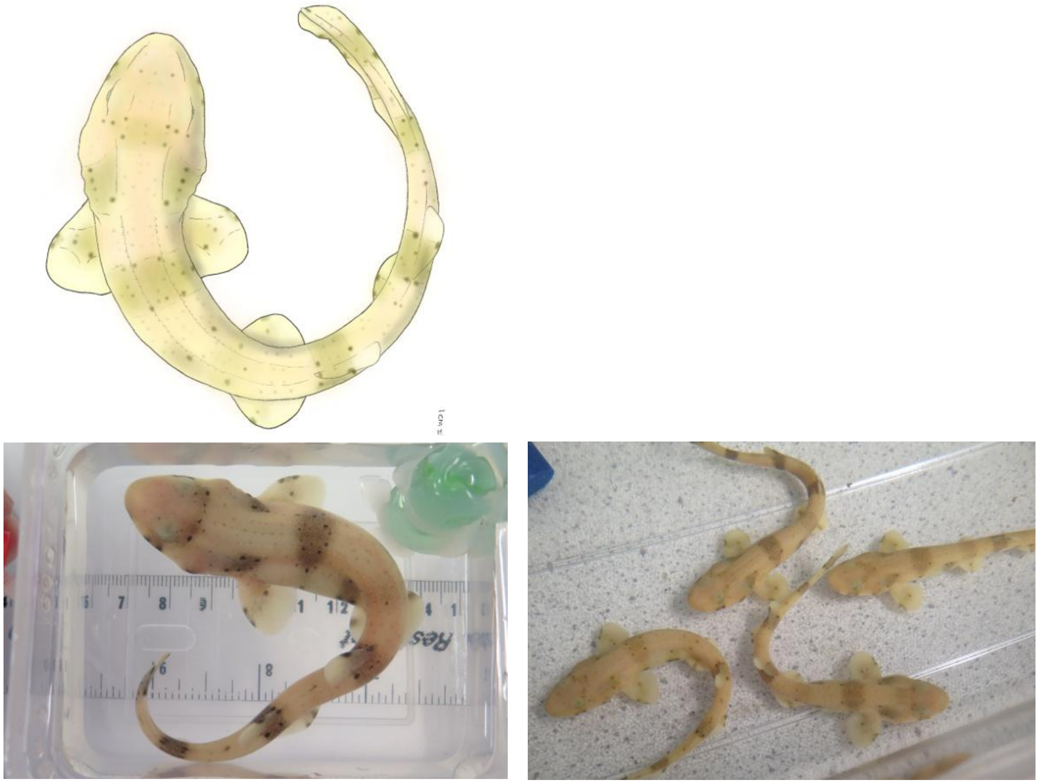

Fig 7B

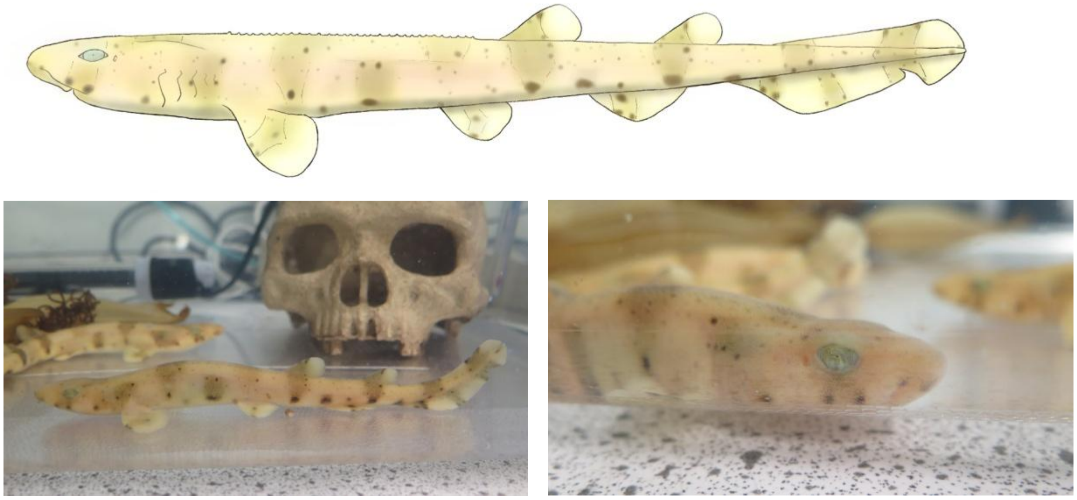

Fig 7C

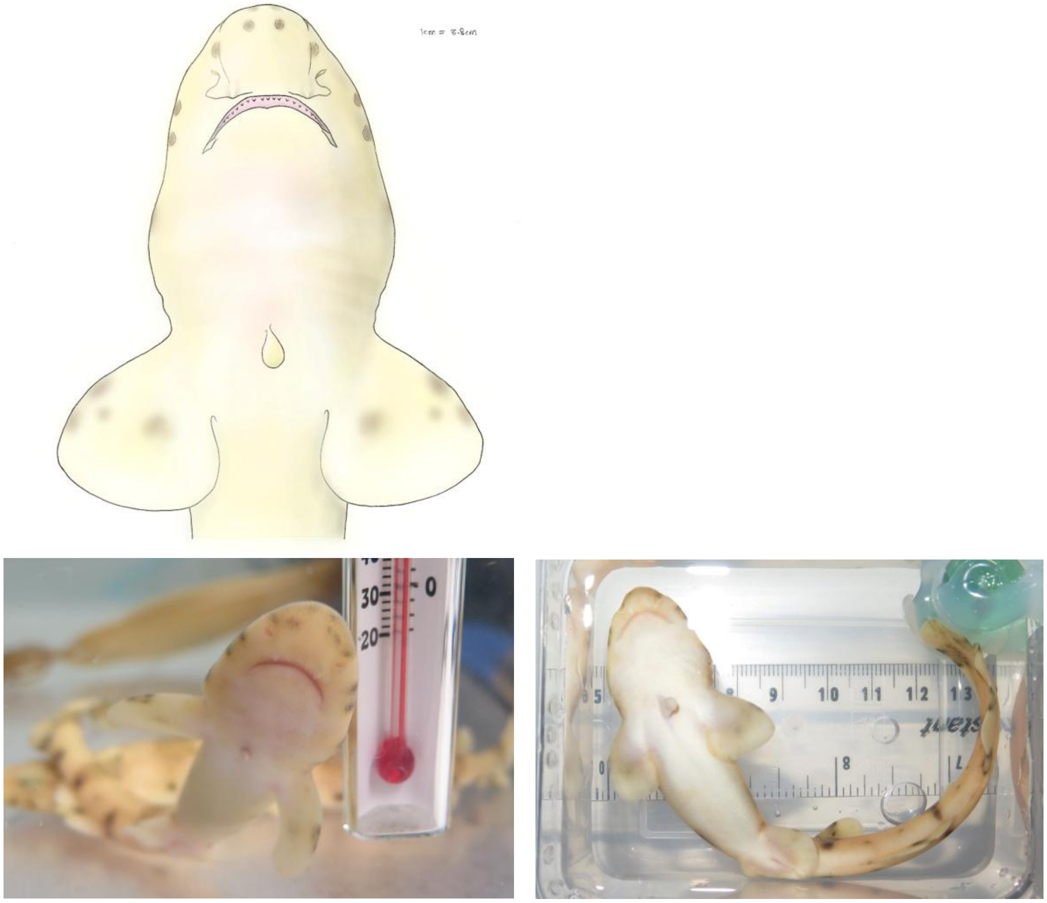

Supplement: S7 File — The inside of the S. stellaris egg case at stage 7. (PDF) [file pone.0206984.s007.pdf]
